# The 1997 North American Interagency Intercomparison of Ultraviolet Spectroradiometers Including Narrowband Filter Radiometers

**DOI:** 10.6028/jres.107.006

**Published:** 2002-02-01

**Authors:** Kathleen Lantz, Patrick Disterhoft, Edward Early, Ambler Thompson, John DeLuisi, Jerry Berndt, Lee Harrison, Peter Kiedron, James Ehramjian, Germar Bernhard, Lauriana Cabasug, James Robertson, Wanfeng Mou, Thomas Taylor, James Slusser, David Bigelow, Bill Durham, George Janson, Douglass Hayes, Mark Beaubien, Arthur Beaubien

**Affiliations:** Cooperative Institute for Research in Environmental Studies, University of Colorado, Boulder, CO 80309, USA; National Institute of Standards and Technology, Gaithersburg, MD 20899-0001, USA; National Oceanic and Atmospheric Administration, David Skaggs Research Bldg., 325 Broadway, Boulder, CO 80303, USA; Atmospheric Sciences Research Center, State University of New York, Albany, 251 Fuller Road, Albany, NY 12203, USA; Biospherical Instruments Inc., 5340 Riley Street, San Diego, CA 92110-2621, USA; National Ultraviolet Monitoring Center, University of Georgia, Athens, GA 30602, USA; Natural Resource Ecology Laboratory, Colorado State University, Fort Collins, CO 80523, USA; Smithsonian Environmental Research Center, P.O. Box 28, Edgewater, MD 21037, USA; Yankee Environmental Systems, 101 Industrial Road, P.O. Box 746, Turners Falls, MA 01376, USA

**Keywords:** environmental monitoring, intercomparison, solar ultraviolet, spectroradiometers

## Abstract

The fourth North American Intercomparison of Ultraviolet Monitoring Spectroradiometers was held September 15 to 25, 1997 at Table Mountain outside of Boulder, Colorado, USA. Concern over stratospheric ozone depletion has prompted several government agencies in North America to establish networks of spectroradiometers for monitoring solar ultraviolet irradiance at the surface of the Earth. The main purpose of the Intercomparison was to assess the ability of spectroradiometers to accurately measure solar ultraviolet irradiance, and to compare the results between instruments of different monitoring networks. This Intercomparison was coordinated by NIST and NOAA, and included participants from the ASRC, EPA, NIST, NSF, SERC, USDA, and YES. The UV measuring instruments included scanning spectroradiometers, spectrographs, narrow band multi-filter radiometers, and broadband radiometers. Instruments were characterized for wavelength accuracy, bandwidth, stray-light rejection, and spectral irradiance responsivity. The spectral irradiance responsivity was determined two to three times outdoors to assess temporal stability. Synchronized spectral scans of the solar irradiance were performed over several days. Using the spectral irradiance responsivities determined with the NIST traceable standard lamp, and a simple convolution technique with a Gaussian slit-scattering function to account for the different bandwidths of the instruments, the measured solar irradiance from the spectroradiometers excluding the filter radiometers at 16.5 h UTC had a relative standard deviation of ±4 % for wavelengths greater than 305 nm. The relative standard deviation for the solar irradiance at 16.5 h UTC including the filter radiometer was ±4 % for filter functions above 300 nm.

Dedicated to the Fond Memory of Douglass Hayes and David Bigelow.

## 1. Introduction

Concern over the effects of changes in solar ultraviolet (UV) radiation on biological ecosystems, humans, and materials has prompted several government agencies in North America to develop UV Monitoring Networks and research programs to address the current and long-term impacts of these changes [1, 2]. Detecting long-term trends in solar ultraviolet irradiance requires accurate measurements of the absolute irradiance, for individual instruments, for an entire network, and between networks [3].

The North American Interagency Intercomparisons of Ultraviolet Monitoring Spectroradiometers are performed near Boulder, Colorado, to assess the ability of spectroradiometers to accurately measure solar ultraviolet irradiance and to compare these results between instruments of different monitoring networks on an annual or bi-annual basis. The first such Intercomparison was held September 19 to 29, 1994; the second Intercomparison was held June 12 to 23, 1995; and the third Intercomparison was held June 15 to 25, 1996. The experimental details and results from these efforts have been described previously [4, 5, 6]. Results from the fourth Intercomparison, held September 17 to 25, 1997, are presented here. This Intercomparison was coordinated by the Optical Technology Division of the National Institute of Standards and Technology (NIST) and the Surface Radiation Research Branch (SRRB) of the National Oceanic and Atmospheric Administration (NOAA). The following agencies and organizations participated: the National UV Monitoring Center (NUVMC) at the University of Georgia which administers the Environmental Protection Agency’s (EPA) UV Network, the National Institute of Standards and Technology (NIST), Biospherical Instruments which administers the National Science Foundation’s (NSF) UV Monitoring Network for Polar Regions, the Smithsonian Environmental Research Center (SERC), the Natural Resource Ecology Laboratory (NREL) of Colorado State University (CSU) which administers the Department of Agriculture’s (USDA) UV Monitoring Network [7], the Atmospheric Sciences Research Center (ASRC) of the State University of New York (SUNY) which represents the USDA monitoring program, and Yankee Environmental Systems, Inc. (YES) which manufacturers a variety of UV instruments used in UV monitoring networks and for atmospheric UV research. A list of attendees is given in Appendix A.

As in previous years, the goal of the Intercomparison was to track the comparison of UV irradiance measured by instruments that are part of UV Monitoring Networks. Unlike other years, there was an emphasis on new prototype instruments for measuring and monitoring of UV irradiance, which precluded a true “blind” comparison of solar irradiance data because of a lack of current participant calibration data for several of the instruments. Synchronized solar scans from each instrument were compared based upon responsivity calibrations performed by NOAA. Measurements of the spectral irradiance responsivity checked the absolute irradiance scales used by the networks and provided a common scale for the synchronized measurements of solar irradiance. As with the other Intercomparisons, these synchronized solar irradiance measurements were the most important aspect of the fourth Intercomparison as they allow assessment of the present limits to which irradiance determined by different instruments can be compared. Instrument parameters characterized included wavelength uncertainty as a function of wavelength, stray-light rejection, the slit-scattering function, bandwidth as a function of wavelength, and spectral irradiance responsivity. A field calibration unit was used for the spectral irradiance responsivity measurements which has been shown to reduce uncertainties of the responsivity for this experiment [8]. Other instruments determined the atmospheric conditions during the Intercomparison, which is useful for correlating these conditions with the measured solar ultraviolet irradiance. A list of all the instruments present at the Intercomparison is given in Table 1.1. Please note the times given in this paper are in Universal Coordinated Time (UTC), 6 h ahead of Mountain Daylight Time, the local time.

## 2. Site Description

The site of the Intercomparison was at the Table Mountain Test Facility (TMTF), a plateau owned by the Federal government approximately 12.9 km north of Boulder, Colorado and 5.6 km east of the Front Range of the Rocky Mountains. This site was chosen because of its good view to the horizon, the presence of laboratory facilities, and the proximity of facility and staff support at both NIST and NOAA in Boulder.

For the synchronized measurements of solar irradiance, the spectroradiometers except the UV-Multi Filter Rotating Shadowband Radiometers (UV-MFRSR) were located on individual concrete pads on the south side of the plateau at latitude 40.125° N, longitude 105.237° W, and elevation 1689 m. The pads were arranged in an east-west line and were 2.4 m square and 12.2 m between centers. The UV-MFRSRs were located at NOAA/SRRB’s site on an elevated platform 50 m to the west of the pads. The highest, and only major, obstruction to the horizon was a peak 5.6 km due west of the pads with a 5.1° angle of inclination. Temporary trailers approximately 30 m south of the pads housed the data acquisition and control computers and equipment for the spectroradiometers. The plateau has a downward slope to the south of the pads, so the tops of the trailers were below the elevation of the pads. At the Intercomparison, pyranometers, pyrgeometers, UVB radiometers, shadowband radiometers and a total sky imager (TSI) were located on the platform. A meteorological tower, recording the temperature, relative humidity, atmospheric pressure, and wind speed and direction at the site was located approximately 90 m north-west of the pads. Finally, a concrete building immediately to the southwest of the platform was used for servicing the instruments and holding meetings. A dome at the western end of the building was covered with a black cloth to eliminate reflections from it to the instruments.

## 3. Instrument Descriptions

For clarity in this paper, spectroradiometers refer to instruments capable of measuring irradiance at particular spectral wavelengths and includes the scanning instruments, the spectrograph instruments, and the narrow-band filter instruments [9]. In total, eleven UV measuring spectroradiometers participated at the Intercomparison. Six scanning spectroradiometers participated at the Intercomparison. These included two Brewer Spectrophotometers,^1^ Model MKIV, serial numbers 101 and 114, operated by the participants from NUVMC at the University of Georgia, which manage the EPA UV Network; a UV Spectroradiometer developed and operated by participants from NIST; a Biospherical Instruments SUV-150 Ultraviolet Spectroradiometer, serial number 11-002, operated by participants from Biospherical Instruments which administer the NSF network; a Smithsonian SR-18 Ultraviolet Scanning Radiometer operated by participants from SERC with 18 defined filter wavelengths, serial number UI; and a prototype U-1000 Spectroradiometer developed and operated by participants from ASRC at SUNY for the USDA UV Network. In addition, five spectral instruments participated at the Intercomparison that have the advantage of measuring a finite number of wavelengths simultaneously. These include two spectrographs that are prototype UV Rotating Shadowband Spectrographs (UV-RSS) where the first is operated by the ASRC for the USDA UV Network, serial number 104, and the second is operated by YES; and three UV Multi-Filter Rotating Shadowband Radiometer (UV-MFRSR), serial numbers 270, 386 and 387 operated by CSU NREL which administer the USDA UV Network. For the remainder of this paper, these instruments will be designated ASRC_RSS, EPA_101, EPA_114, NIST, NSF_SUV, SERC, USDA_U1K, USDA_270, USDA_386, USDA_387, YES_RSS where the acronym conveys the UV Network or manufacturer followed by the instrument type or serial number. Table 3.1 lists the characteristics of each instrument, and descriptions are given below. The two Brewer spectroradiometers, the SERC multi-filter radiometer, and the UV-MFRSRs were described in detail earlier [4, 5, 6, 10] and therefore only a cursory description is given below. The other instruments are given a more detailed description in this paper or an appropriate reference is cited.

### 3.1 Brewer Spectrophotometer

Two Sci-Tec Brewer spectrophotometers (Model MKIV) participated at the Intercomparison that measure total solar ultraviolet irradiance from 286.5 nm to 363 nm and total column O_3_, SO_2_, and NO_2_ from both direct sun and zenith sky measurements at specific wavelengths. A right-angle prism directs light from one of several sources, either internal calibration lamps, the sky, or a Teflon diffuser, along the optical path. This path contains apertures, filters, and lenses that focus the light onto the entrance slit of a single-grating modified Ebert-type monochromator.

The exit slit focal plane of the monochromator contains six slits, five for selecting the wavelengths for determining the total column amounts and one for wavelength calibration. A slotted cylindrical slit-mask in front of the exit slit plane serves as the wavelength selector. The nominal bandwidth, set by the exit slits, is 0.6 nm. For the Model MKIV Brewer spectrophotometer, the diffraction grating operates in third order for UV spectral scans and O_3_ and SO_2_ measurements and second order for NO_2_ measurements. Light from the exit slit passes through a lens and a filter before focusing onto the cathode of a photomultiplier tube (PMT). The photon pulses from the PMT are amplified, discriminated, and divided by the slit-mask cycle before being transmitted to the counter. For wavelengths shorter than 325 nm the MKIV model uses a NiSO_4_ filter sandwiched between two Schott UG-11 filters, and a single UG-11 filter for longer wavelengths.

The wavelength of the monochromator in terms of micrometer steps of the instrument is determined at the factory from the wavelengths of Hg emission lines. The wavelength registration of the monochromator is periodically checked and adjusted throughout a day by scanning the micrometer forward and backward about the 302.3 nm line from the internal Hg calibration lamp. The EPA Network uses a set of lamps, housing, and power supply furnished by the manufacturer for stability checks. These are 50 W quartz-halogen lamps mounted horizontally 5 cm above the diffuser in a housing and operated at a constant 12 V.

### 3.2 Biospherical Ultraviolet Spectroradiometer

**Fig. 3.1:**
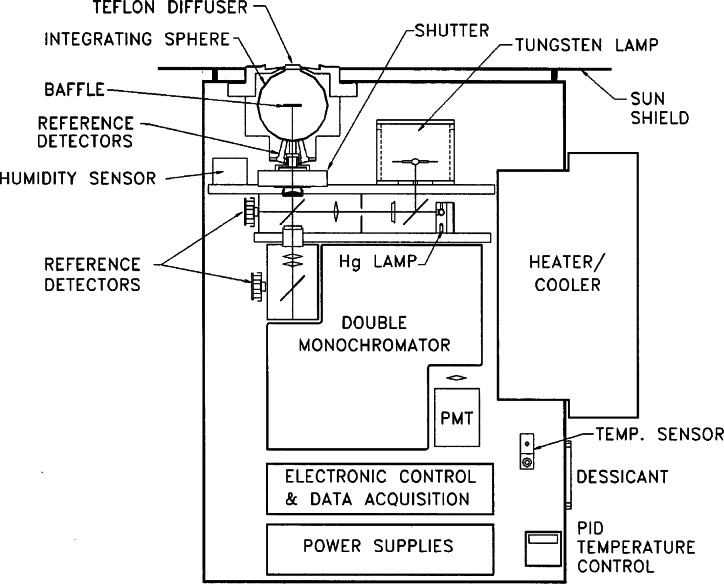
Schematic diagram of Biospherical Instruments’ SUV-150 spectroradiometer (NSF_SUV).

The Biospherical SUV-150 11-002 Ultraviolet Spectroradiometer is a 150 mm f/4.4 Czerny-Turner double monochromator that employs a grating blazed at 240 nm with 2400 grooves per millimeter. The instrument has a nominal bandwidth of 0.7 nm and typically scans from 280 nm to 600 nm in 0.2 nm steps, taking approximately 16 minutes to complete a scan. A schematic of the SUV-150 is given in Figure 3.1. The SUV-150 utilizes a quartz window with a vacuum-formed Teflon diffuser at the entrance port of an integrating sphere. The diffuser is heated to minimize ice and snow buildup. The monochromator is coupled to a 9-stage dynode R2371 PMT mounted in a shielded temperature regulated semi-hermetic enclosure. The temperature is maintained within ±1 °C by a thermoelectric heater/cooler, driven by a PID controller. The instrument is fully automated and weatherproofed for use in extreme conditions.

The SUV-150 utilizes two internal lamps for wavelength and intensity calibration. The lines from a Hg emission lamp are scanned each day and are used to register the monochromator at the 253.7 nm and 296.7 nm lines and determine the wavelength transfer function. In addition, if needed, several manual Hg lamp scans can be performed by an operator. The other internal calibration source is a 45 W quartz-tungsten-halogen lamp for determining the spectral irradiance responsivity of the instrument. The responsivity is determined in a two-step process since the high voltage of the PMT is variable. The spectral irradiance scale is determined by a 200 W DXW-type quartz-tungsten-halogen lamp mounted horizontally 50 cm above the diffuser in a custom enclosure. A spectral scan of this lamp at a fixed high voltage is followed by a spectral scan of the internal 45 W lamp at the same high voltage. This serves to calibrate the internal lamp, and spectral scans of this same lamp at different high voltages determines the responsivity under the particular operating condition.

### 3.3 NIST Ultraviolet Scanning Spectroradiometer

**Fig. 3.2:**
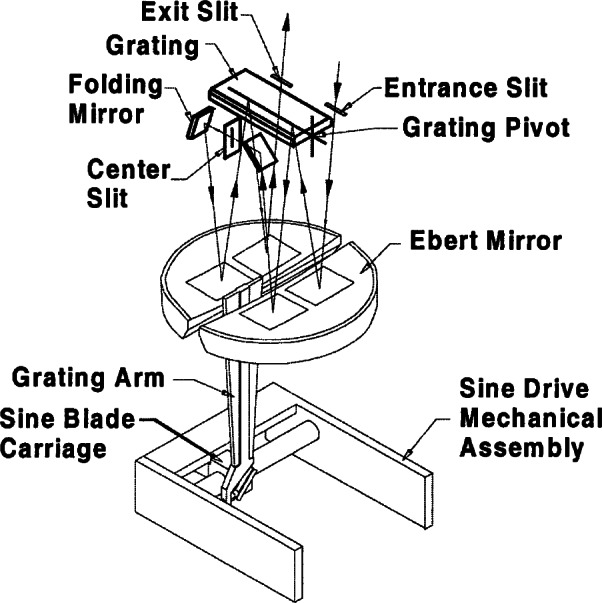
Schematic diagram of the optical path of the NIST spectroradiometer.

The NIST Ultraviolet Spectroradiometer is one of several double monochromators that were designed and built for NIST in the early 1980s [11, 12, 13]. The design uses a 1/8 m Fastie-Ebert monochromator with mirror and grating blanks of Zerodur to assure thermal stability. A schematic diagram of the optical path is given in Fig. 3.2. This f/5 monochromator utilizes a single rectangular grating in a double pass configuration in order to achieve a high degree of wavelength accuracy. In the first monochromator chamber, one end of the rectangular grating is used and in the opposite chamber the other end of the grating is used. Therefore, the double monochromator uses a single grating shaft to change the wavelength in both chambers, minimizing the wavelength uncertainty. This instrument is modular with two Fastie-Ebert monochromators that can be interchanged depending on the measurement project and is easily aligned with the fore-optics and control and drive electronics. The two Fastie-Ebert monochromators employ blazed holographic gratings at 250 nm with 3600 and 1800 grooves per millimeter, respectively. The former grating is used during the Intercomparison with a wavelength range of 290 nm to 325 nm. The slit-widths are fixed and are set for a bandwidth of 0.8 nm.

**Fig. 3.3:**
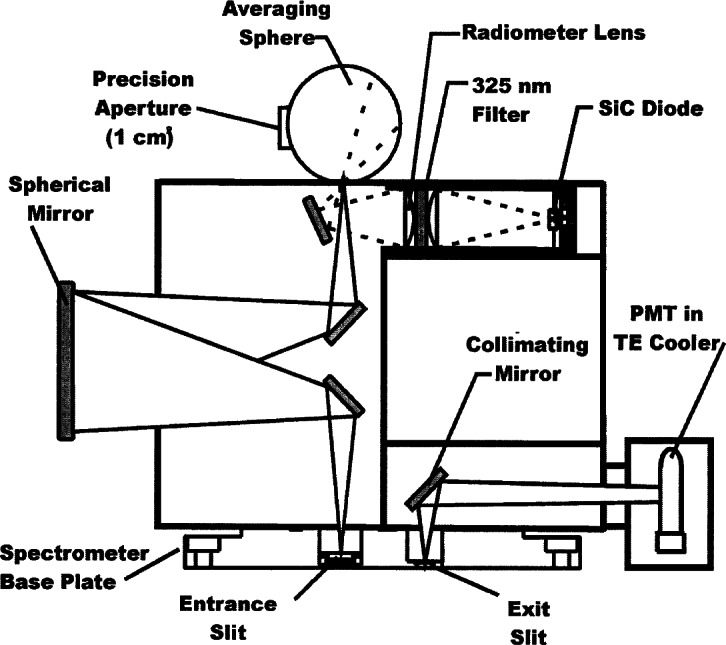
Schematic diagram of the entrance and detector relay optics of the NIST spectroradiometer.

A separate compartment houses the relay optics and attachment of the detectors and entrance fore-optics. A schematic of the entrance and detector relay optics for the spectroradiometer are shown in Fig. 3.3 where the entrance optics of the monochromator have been rotated 90° for illustrative purposes. The entrance fore-optics consists of either an averaging sphere or a transmitting diffuser assembly. The 52 mm averaging sphere has a pressed polytetrafluoroethylene (PTFE) coating. A precision aperture (1.004 cm^2^) is located at the averaging sphere entrance port. An interference filter radiometer views the exit port of the irradiance fore-optics, slightly off-axis from the monochromator viewing geometry, and is used to monitor the source stability. The entrance relay optic is a doubly folded spherical mirror with unit magnification. A small spherical mirror is used as a collimating relay optic from the exit port to the photomultiplier (PMT) detector. Two side-window PMTs have been used: a “solar blind” CsTe PMT with low responsivity at wavelengths greater than 310 nm and a bi-alkali PMT with sensitivity to about 650 nm. The former was used at the Intercomparison. The PMT is cooled with a thermoelectric cooler and the PMT signal is measured with photon-counting electronics. The instrument is equipped with temperature sensors at the detectors and in the monochromator. An external 1000 W tungsten halogen spectral irradiance lamp for responsivity calibrations is operated in a field unit [5, 8]. The current from the power supply was maintained at the desired value by the control computer. The instrument used at the Intercomparison is not weatherproof and is built for automatic operation.

### 3.4 Smithsonian Ultraviolet Scanning Radiometer

**Fig. 3.4:**
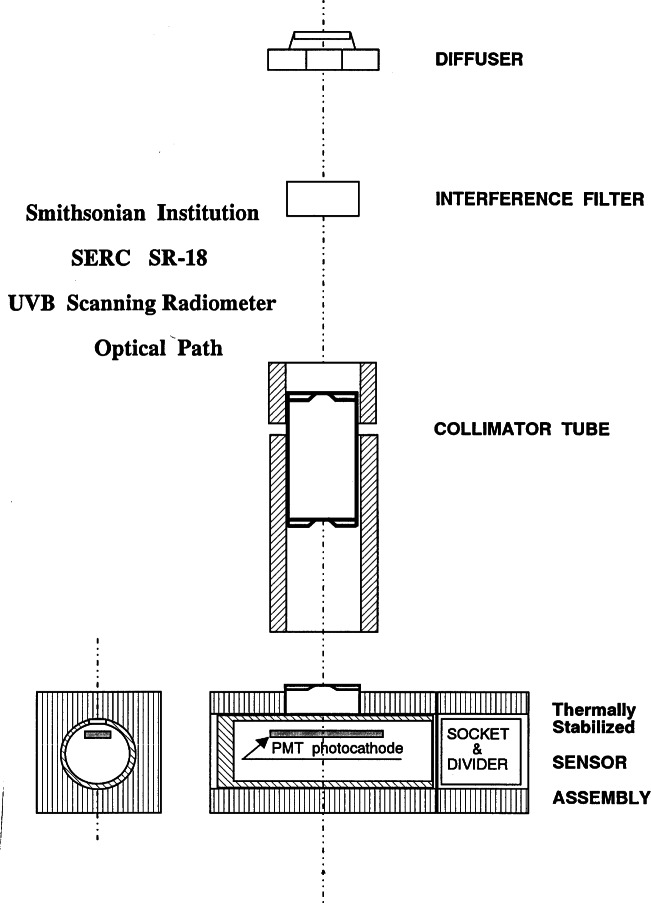
Schematic diagram of the optical path of the SERC UV Scanning Multi-filter Radiometer.

The Smithsonian SR-18 Ultraviolet Scanning Radiometer, serial number UI, measures total solar ultraviolet irradiance at fixed wavelengths selected by 18 interference filters from 290 nm to 324 nm with nominal 2 nm bandwidths. A schematic diagram of the optical path is given in Fig. 3.4. The nominal and actual filter center wavelengths, bandwidths, and maximum transmittances of unit UI are given in Table 3.2. The filters are located on a filter wheel, which rotates at 15 rev/min underneath a Teflon diffuser. Light from the diffuser passes through a filter and is detected by a solar-blind PMT (Hamamatsu R1657) operating in current mode at 20 °C. The output current is converted to voltage and 14 samples averaged for one minute for each filter, i.e., one spectrum is taken every 4.3 s. The spectral irradiance responsivity is determined at SERC by operating a calibrated 1000 W FEL-type quartz-halogen lamp in the horizontal position centered 50 cm above the diffuser.

### 3.5. USDA/ASRC U1000 Ultraviolet Spectroradiometer

**Fig. 3.5:**
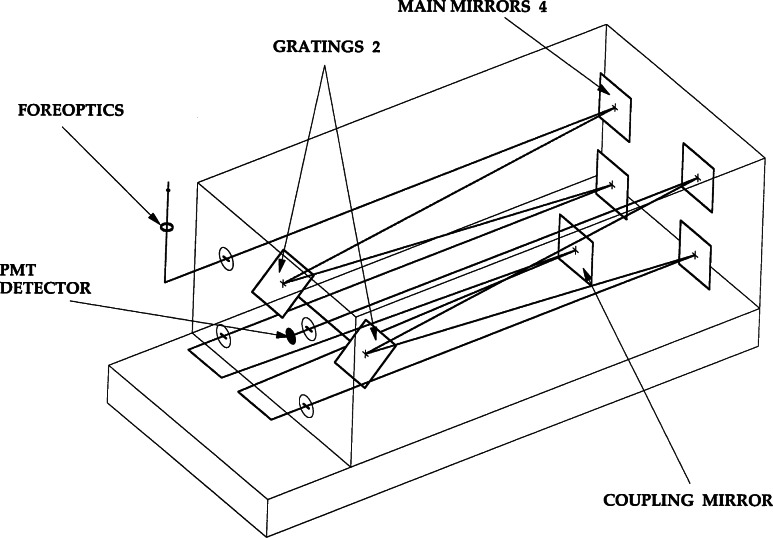
Schematic diagram of the optical path of the USDA_U1K spectoradiometer.

The USDA reference Ultraviolet Spectroradiometer is a recent prototype design built by ASRC and Instruments SA for use in the USDA UV/Biosphere Network. The monochromator is a 1-meter double Czerny-Turner design with an ion-etched holographic grating operated in first order with 3600 lines per mm. The optical path is given in Fig. 3.5. The detector is a Hamamatsu R2371-02 PMT with an ASRC-developed dual-threshold photon counting system with a maximum synchronous counting capability of 600 MHz. The wavelength range extends from 290 nm to 410 nm. The nominal bandwidth is 0.1 nm and the instrument can operate with a step-size of 0.0005 nm/step and a FWHM of 0.01 nm, preserving a triangular slit function and commensurate throughput if carefully aligned. The instrument is capable of an out-of-band rejection ratio of approximately 10^−10^. The measurements at short wavelengths are limited by dark signal and integration time rather than stray light. At wavelengths greater than 295 nm, the uncertainty in the signal given by Poisson statistics is less than 5 %, and less than 1 % beyond 299 nm. The diffuser material is PTFE with a diameter of 2.54 cm. Because this instrument is designed for use in the USDA Monitoring Network, the instrument is both automatic and weatherproof.

Calibrations are performed with an external 1000 W FEL type lamp by the Central Ultraviolet Calibration Facility (CUCF). The instrument also has two identical 20 W halogen lamps in the fore-optics supplied by a non-precision constant voltage source. Later instruments deployed in the network will use a quality constant-current source. In normal operation one of the two identical lamps is designated the working lamp and is automatically checked each night, the other lamp is burned infrequently, typically once every 2 weeks. At this Intercomparison both were measured each night. Wavelength calibration is completed with an internal Hg low-pressure emission lamp.

### 3.6. USDA/ASRC Ultraviolet Rotating Multi-Filter Shadowband Radiometer

The UV-MFRSR uses independent interference filter-photodiode detectors and an automated rotating shadowband to measure the direct-normal, total-horizontal, and diffuse-horizontal ultraviolet solar irradiance at seven wavelengths [14]. The instrument is manufactured by Yankee Environmental Systems following a similar design developed at the Atmospheric Science Research Center (ASRC) at SUNY, Albany. Three of these instruments, units 270, 386, and 387, participated at the Intercomparison. The prototype unit 270 participated at a previous Intercomparison but has undergone several modifications since then.

The diffuser used to collect the incident radiant flux and the detectors that measure it are located in the sensor head of the detector assembly. The diffuser is a thin walled Teflon integrating cavity protruding above the top of the head and surrounded by an artificial horizon to improve the angular response of the instrument. Two diaphragms of frosted WG-280 glass in the integrating cavity act as transmission diffusers. Light exiting the bottom of the diffuser is incident on a hexagonal array of photodiodes with a seventh photodiode in the center of the array, all with interference filters. The nominal and actual filter center wavelengths and bandwidths of both units are given in Table 3.3. The interior of the head is thermally insulated and has a thermostatic electrical heater that holds the temperature at 45 °C. The shadowband assembly has been described in a previous Intercomparison reference [6]. Measurement of the total-horizontal and diffuse-horizontal ultraviolet solar irradiance sequence occurs three times per minute. The instrument can average over selected time intervals and one minute averages were used for the Intercomparison.

### 3.7. Ultraviolet Rotating Shadowband Spectrograph (UV-RSS)

Two Ultraviolet Rotating Shadowband Spectrographs (UV-RSS) participated at the Intercomparison, one operated by Yankee Environmental Systems and the second operated by the Atmospheric Sciences Research Center (ASRC). The two spectrographs had comparable designs but the following description is for the ASRC UV-RSS which was the first prototype developed at the Atmospheric Sciences Research Center, SUNY-Albany from its predecessor, the RSS [15]. The automated shadowband method [14] allows the instrument to measure quasi-simultaneously diffuse-horizontal and total-horizontal spectral irradiance, guaranteeing identical bandpass and calibration coefficients for the separated components. However, this first UV-RSS prototype was operated without the shadowband attachment during the Intercomparison and measured only the total horizontal irradiance required by the Intercomparison protocol.

**Fig. 3.6:**
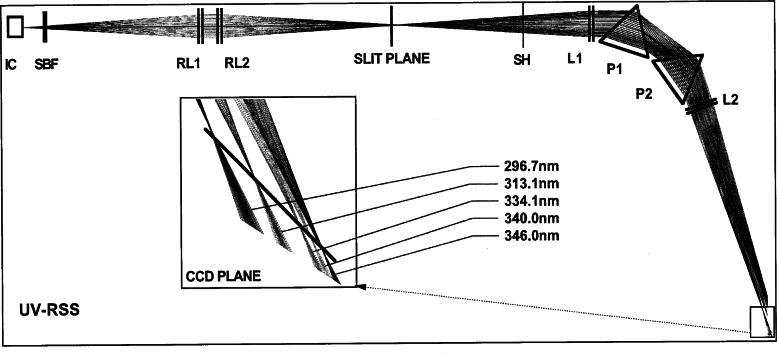
Schematic diagram of the optical path of the ASRC_RSS spectrograph.

The optical diagram of the UV-RSS is shown in Fig. 3.6. The fore-optics consists of the diffuser/plug integrating cavity (IC) walls from PTFE, and the internal plug forming the bottom surface of the integrating cavity is Spectralon. The design was empirically optimized for Lambertian response and throughput. The exit aperture of the integrating cavity is a 0.300 mm × 3.000 mm rectangle. The solar-blind filter (SBF) is made of NiSO_4_ crystal sandwiched between Schott UG-5 glass elements.

The relay lenses (RL1, RL2) are made from UV-grade fused silica. Their purpose is twofold: to match the spectrograph’s numerical aperture and to reduce the dynamic range of the incoming signal. The lenses are an aperture stop, reducing stray light in the spectrograph. Their chromatic aberration is used intentionally to reduce the throughput at longer wavelengths; the relay system is focussed to maximize throughput at the 296.7 nm Hg line.

Lenses (L1, L2) and prisms (P1, P2) of the dual-prism spectrograph are also made of the UV-grade fused silica with all primary surfaces polished to 20-10 scratch/dig quality and with multi-layer broadband anti-reflective UV coatings on all primary surfaces. The bases of the prisms remained uncoated to reduce the cost. The throughput of the spectrograph is controlled by the f-number (f/7.5) and the size of the entrance slit (0.050 mm by 2.000 mm).

This prototype used a relatively noisy metal-oxide-semiconductor (MOS) linear detector array from Hamamatsu with 512 pixels (0.025mm × 2.500mm). This is far from optimum for the UV, but was available and well tested at the time. Subsequently, both standard and UV-RSS instruments use astronomical-grade 1024 × 256 CCD arrays. The wavelength range is determined by the size of the detector array and magnification of the spectrograph optics. In the UV-RSS, pixel 1 corresponds to 295.8 nm and pixel 512 to 348.8 nm. Later UV instruments with the 1024 × 256 CCD arrays span the range 288 nm to beyond 360 nm. The exposure for the UV-RSS was set to 10 s. Each measurement cycle consisted of one open-shutter (SH) 10 s exposure followed by one closed-shutter 10 s exposure to measure dark signal. After adding the readout time and data transfer time, the total horizontal irradiance at 512 adjacent wavelengths was acquired every 27.12 s.

The fore-optics and spectrograph are dry-air purged and temperature stabilized. The former prevents damage to optics and the detector array and the latter improves dark signal and wavelength stability. Wavelength shifts are driven chiefly by the temperature of the prism, thermal stress on the fore-optics, and the air density within the spectrograph. No provision was made on this prototype to control the latter.

**Fig. 3.7:**
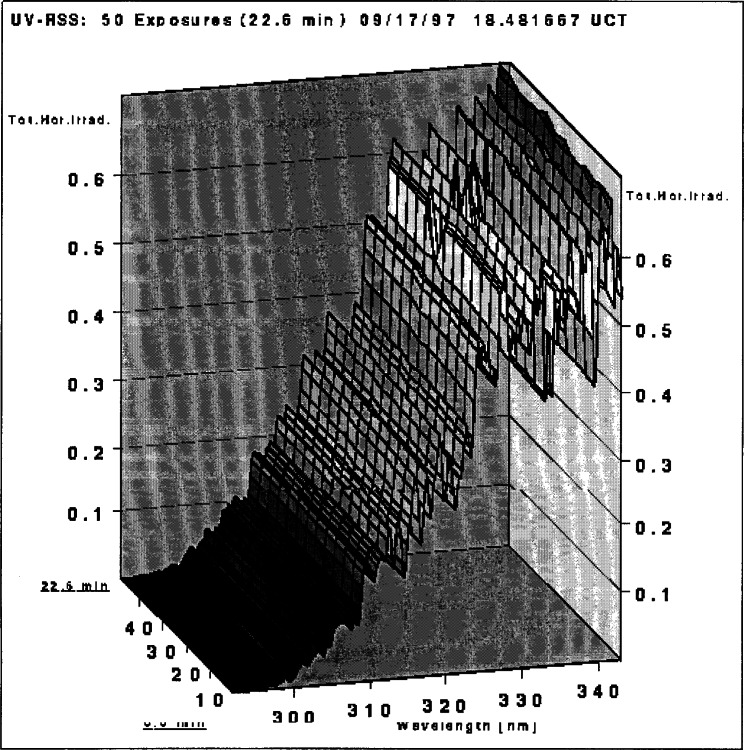
Solar irradiance surface measured by the ASRC_RSS spectrograph. The pseudo-scan is synthesized from a diagonal cut of the irradiance surface.

The Intercomparison protocol was designed for the common scanning instruments that acquire spectra sequentially. The RSS simultaneously acquires all 512 spectral elements every 27.12 s. Therefore, to obtain comparable results, given time-varying irradiance, pseudo-scans were synthesized from the assembly of RSS exposures to correspond to the scanning instruments’ irradiance measurements. On average 50 sequential exposures were used to extract data for one pseudo-scan. The pseudo-scan was synthesized from a diagonal cut of the irradiance surface depicted in Fig. 3.7. The 512 points on the diagonal were subsequently interpolated into the grid of 531 points starting at 295.8 nm every 0.1 nm. The remaining 98 % of the data points produced by the RSS were discarded as they did not coincide with the time-wavelength points specified by the Intercomparison protocol.

## 4. Atmospheric Conditions

Weather conditions for the Intercomparison (Julian day 257 – 267) were mostly unfavorable with periods of torrential downpours. During the determination of the slit function, stray-light, and wavelength accuracy the weather was moderately favorable with predominately clear skies in the morning and increasing cloudiness in the afternoons with drizzle (Julian day 257 – 260). During most of the days of the responsivity determinations and synchronized scans, the skies were completely overcast, with intermittent rain and downpours. Fortunately, the weather cleared for the final day (267) of the synchronized scans providing an almost cloud-free day.

The temperature, relative humidity, barometric pressure, and wind speed and direction were recorded at the site of the Intercomparison by the meteorological instruments listed in Table 1.1. During the first few days of the Intercomparison (days 257 to 261), the temperature ranged from 15 °C in the early morning to nearly 30 °C in the late afternoon. After the rain clouds moved into the area, the temperature ranged from an average of 5 °C in the early morning to approximately 12 °C in the late afternoon (days 262 to 266). On the final day (267) of the synchronized scans when the storm cleared, the temperature varied from 5 °C in the early morning to nearly 25 °C in the late afternoon. The relative humidity remained around 30 % prior to the storm, increased to over 100 % during the storm, then dropped from 80 % to 40 % during the clear sky day of 267. The barometric pressure ranged from 83.2 kPa (day 261) to a maximum of 84.4 kPa (day 263).

**Fig. 4.1:**
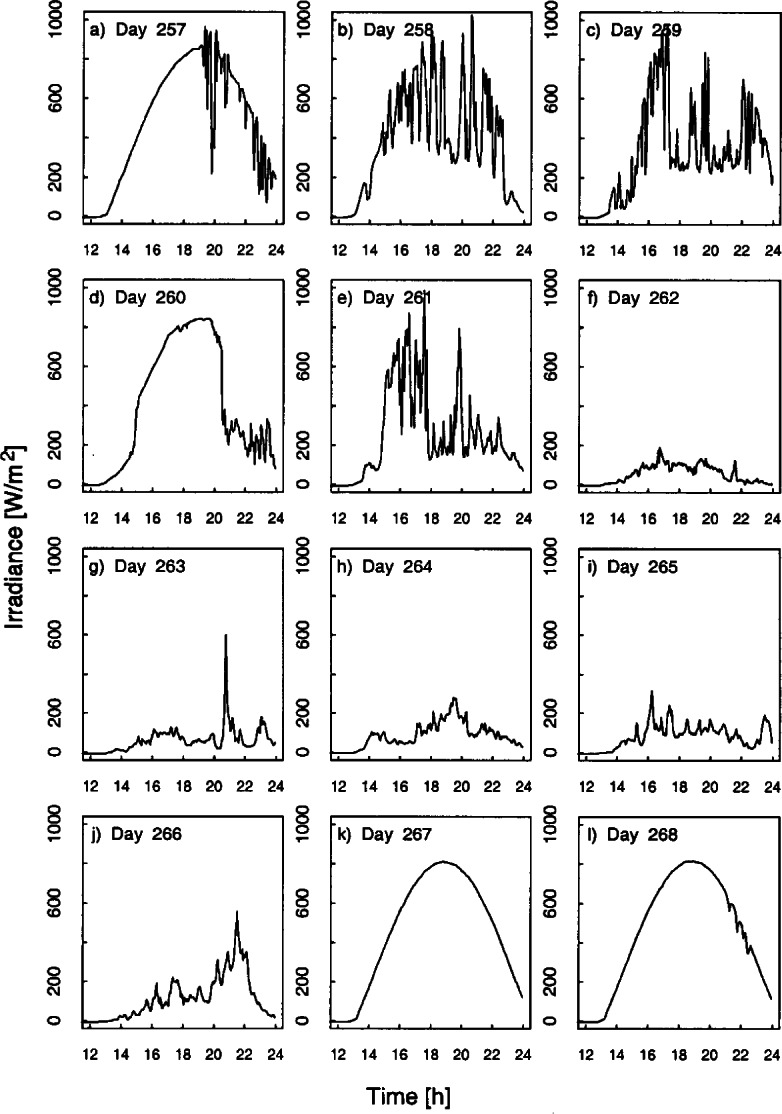
Total horizontal irradiance as a function of time from a solar pyranometer on the days indicated in the panels. Solar noon occurs at approximately 19.0 h UTC.

A set of broadband radiometric instruments, listed in Table 1.1, were located on the test facility platform and made continuous measurements concurrently with the Intercomparison. Results from one solar pyranometer are shown in Fig. 4.1, where the irradiance is plotted as a function of time for each day. This solar pyranometer measured total horizontal irradiance from 280 nm to 3000 nm. The clear morning skies and increasing afternoon cloudiness on day 257 are evident in Fig. 4.1. On day 260, inclement weather moved into the area and remained through day 266 making conditions difficult for determining the responsivity and performing synchronous solar scans. On the night of day 266, the weather cleared and the sky was clear virtually the entire next day.

**Fig. 4.2:**
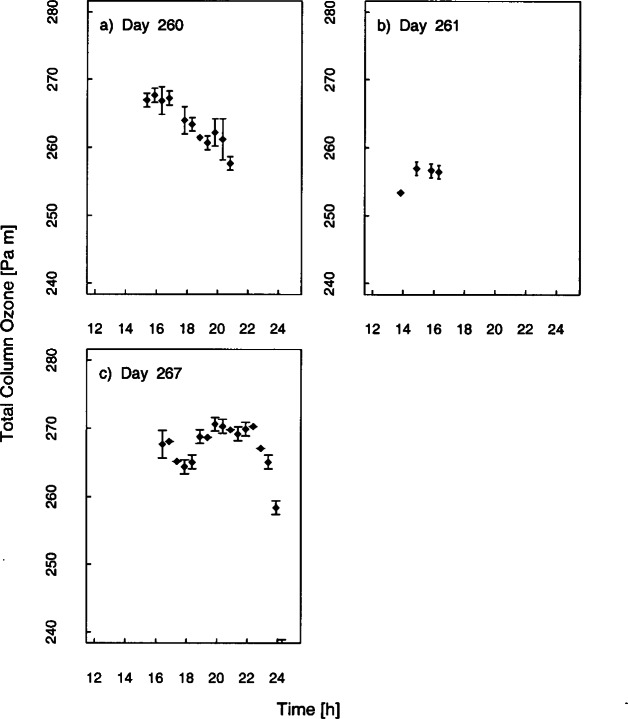
Total column ozone as a function of time on the days indicated in the panels as determined by the Brewer spectroradiometer, serial number 114. The vertical bars are the standard deviations of the values.

The EPA Brewer, number 114, determined total column ozone throughout the Intercomparison from measurements of the direct solar beam. The results are shown in Fig. 4.2, where the total column ozone is plotted as a function of time for each day. The vertical bars are the standard deviation of each value. The total column ozone averaged 264 ± 3 Pa·m (260 matm·cm) on day 260. The sky was too overcast from mid-day 261 through day 266 for accurate measurements. For the one clear sky day during the Intercomparison, day 267, the total column ozone averaged 267 ± 3 Pa·m (264 matm·cm).

## 5. Instrument Characterizations

The spectroradiometers were characterized for the parameters that most affect their ability to accurately measure solar ultraviolet irradiance, and which did not require elaborate experimental equipment or techniques. Therefore, the slit-scattering function, stray-light rejection, wavelength uncertainty, bandwidth, and spectral irradiance responsivity were determined. All of the characterizations were performed outdoors for the instruments on the pads. Previous Intercomparisons demonstrated the need to perform the characterizations outdoors to eliminate moving the instruments after the characterizations. Since detailed mathematical discussions of the characterization techniques based upon a simple measurement equation have been given previously [4], they will not be repeated here. Please note that the YES_RSS instrument had instrumental difficulties early in the campaign and therefore the slit-function and stray-light characterization tests were not performed on this instrument.

### 5.1 Slit-Scattering Function and Stray-Light Rejection

#### 5.1.1 Experimental Procedure

An Omnichrome Model 3056 HeCd laser with a single line at 325.029 nm and a nominal power of 18 mW was used to determine both the slit-scattering function and the stray-light rejection of the instruments. The laser was mounted on a tripod, and a box with a hole was placed on top of the instrument. The output of the laser was directed through the hole directly onto the diffuser. The beam diameter was approximately the same diameter as the diffusers. The outdoor measurements were performed at twilight and in the evening to minimize the background signal from the sky.

High-resolution spectral scans were performed near 325 nm to obtain the bandwidth of the instrument, centroid of the line, and shape of the slit-scattering function near its peak. Lower-resolution spectral scans were performed across the entire wavelength ranges of the instruments to obtain the full slit-scattering function. For the SERC instrument, the signals were measured for 4 min. The instruments were configured so that the maximum signal did not saturate the PMT. For the EPA instruments, this involved using an internal neutral-density filter for the high-resolution scans, and then removing the filter from the optical path for the low-resolution scans. A lower-resolution scan was also performed with the laser beam blocked to check for stray light from sources other than the laser. There were no signals greater than the dark signal for any of the instruments.

#### 5.1.2 Data Analysis

The bandwidth of the instrument is defined here as the full-width-at-half-maximum (FWHM) from a high-resolution spectral scan of a laser line or a singlet lamp emission line. Linear interpolation is used to find the wavelengths at which the signal is one-half that of the peak. The bandwidth is then the difference between these two wavelengths.

The centroid method is used to estimate the wavelengths of laser lines and lamp emission lines. The centroid *C* from a high-resolution scan is given by

$$C=\sum_{i}S_{i}\lambda_{i}/\sum_{i}S_{i},$$ (5.1)

where *i* indexes the signals *S_i_* and wavelengths *λ_i_*, respectively, of those signals greater than 0.1 of the peak signal. Although baseline subtraction is not important for calculations of the centroid of laser lines because the light is monochromatic, to maintain consistency with the bandwidths determined by lamp emission lines, baseline subtraction was performed for spectral scans of laser light. A description of the procedure is given in Sec. 5.2.3.

For the high-resolution scans, normalization of the signals by the peak signal was straight-forward because there is no saturation of the signal. For the low-resolution scans, the peak signals obtained in the high-resolution scans and the optical densities of the filters were used to calculate the peak signals for the scans without the neutral-density filters. The optical density at 325 nm of a neutral-density filter was determined from the common wavelengths at which signals were measured for scans both with and without the filter.

The peak signals obtained in the high-resolution scans were used to normalize the signals from the low-resolution scans for the ASRC_RSS, NIST, NSF_SUV, and USDA_U1K instruments since there was no saturation. The peak signal for the SERC instrument was not as readily known because there is no filter centered at 325 nm. Therefore, the peak signal for each filter was obtained from the measured signal of the filter centered at the longest wavelength that did not saturate. These peak signals were calculated by dividing the measured signal from the filter centered at 320.65 nm by the transmittance of that filter at 325 nm and multiplying by the peak transmittance of each filter.

#### 5.1.3 Results and Discussion

**Fig. 5.1:**
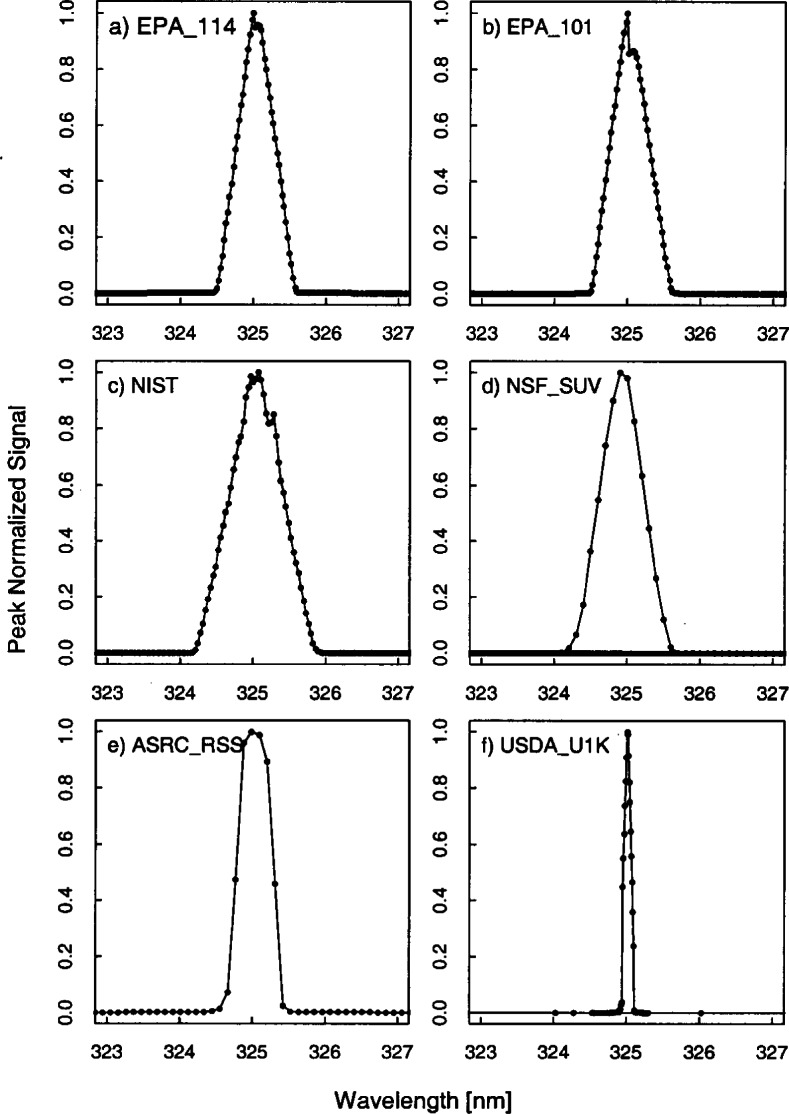
Peak-normalized signal as a function of wavelength from high-resolution spectral scans of the 325.029 nm line from a HeCd laser for the instruments indicated in each panel, demonstrating the slit-scattering functions. The shift in signal at 325 nm in the EPA_101 and EPA_114 is due to several filter changes at this wavelength.

The bandwidths of the instruments and the centroids of the laser line are most useful when included with those values obtained from the scans of the Hg, Cd, and Zn lamps. Therefore, the results from these determinations are shown in Figs. 5.3 and 5.4 in the next section, but to summarize, the bandwidths using the 325 nm line of the HeCd laser are close to the nominal values giving 0.53 nm for the ASRC_RSS instrument, 0.58 nm for EPA_101, 0.58 nm for EPA_114, 0.85 nm for the NIST instrument, 0.70 nm for the NSF_SUV instrument, 0.11 nm for the USDA_U1K instrument, and 0.61 nm for the YES_RSS instrument. These are given in Table 5.1 and the slit-scattering functions are given in Fig. 5.1. The shift in the signal at 325 nm for the EPA_101 and EPA_114 is due to several filter changes at that wavelength. From Fig. 5.1, the slit-scattering functions of the EPA_101, EPA_114, NIST, and USDA_U1K instruments are nearly triangular and symmetric about the peak wavelength. The wings of the slit function measured with the HeCd laser are chiefly determined by bulk and surface scattering of the spectrograph optics. The slit-scattering function was not determined for the YES_RSS instrument due to YES_RSS instrumental problems at the time.

**Fig. 5.2:**
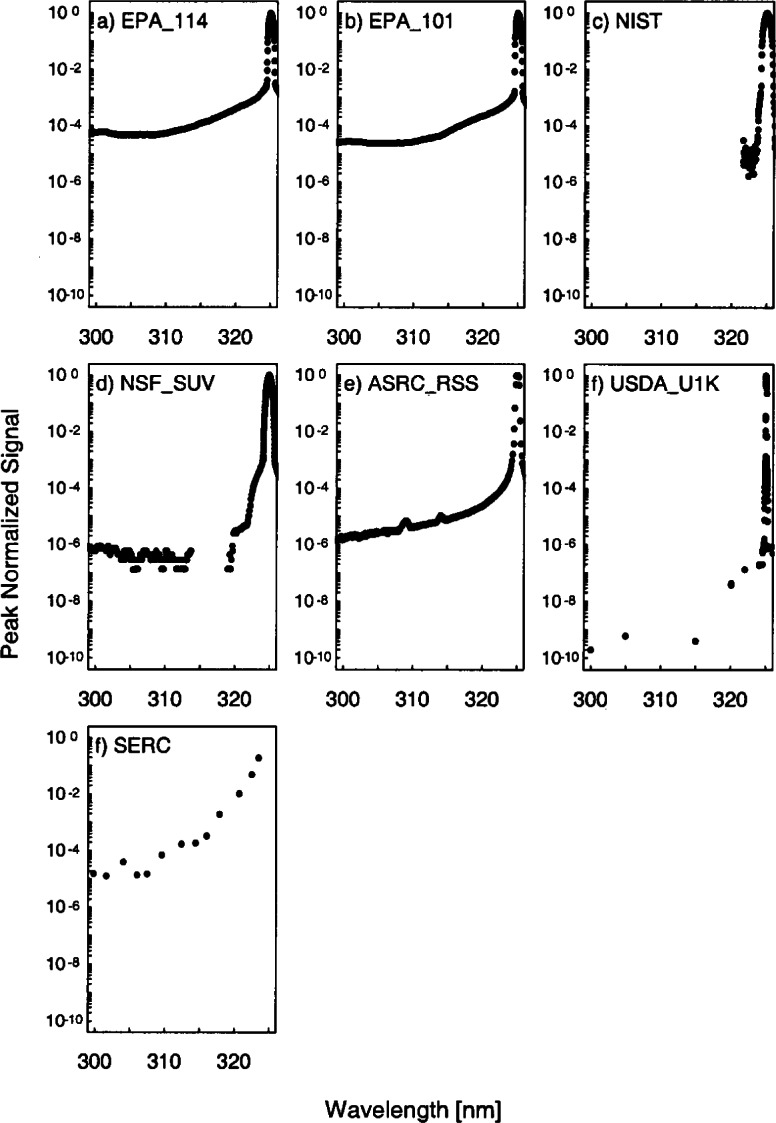
Peak-normalized signal as a function of wavelength from low-resolution spectral scans of the 325.029 nm line from a HeCd laser for the instruments indicated in each panel, demonstrating the stray-light rejections.

The stray-light rejection of each instrument is shown in Fig. 5.2 and is determined from high- and low-resolution scans where the peak-normalized signal is plotted as a function of wavelength. The stray-light rejection is reported for 300 nm in Table 5.1 except for the NIST instrument, which is reported at 320 nm. The stray-light rejection of 3 × 10^−5^ and 6 × 10^−5^ for instruments EPA_101 and EPA_114 is reasonable for Brewer instruments because they are single-grating instruments. For the NIST and the NSF_SUV instruments, the measurement of the stray-light rejection is probably limited by the dynamic range of the detector and can only be reported as better than 10^−5^ and 10^−6^, respectively. The ASRC_RSS instrument had a stray-light rejection of approximately 2 × 10^−6^. The USDA_U1K instrument is also a double monochromator and has the greatest dynamic range with a measured stray-light rejection of better than 2 × 10^−10^. The stray-light rejection of the SERC instrument of approximately 2 × 10^−5^ is also reasonable for interference-type filter instruments. The features at 309 nm and 314 nm for the ASRC_RSS instrument result from recycled rays within the prisms that have undergone three internal reflections. Their location is wavelength dependent. Developers of the ASRC_RSS instrument (ASRC SUNY) have suggested that the magnitude of these features could be further reduced with an anti-reflective coating for the prism bases.

### 5.2 Wavelength Uncertainty

#### 5.2.1 Introduction

Characterizing the instruments in terms of their response to light from Hg, Cd, and Zn emission line lamps is somewhat more complex than was the case for a HeCd laser because there is a continuum in addition to the lines, and because there can be unresolved multiple lines. However, it is useful because it yields information at several wavelengths about the bandwidth and the wavelength dependence and wavelength uncertainty of the instruments. The wavelength uncertainty is especially important in the UV-B region of the solar spectrum (280 nm to 315 nm) because the irradiance at the Earth’s surface changes rapidly with wavelength and therefore a small uncertainty in wavelength can translate into a large uncertainty in irradiance.

A distinction needs to be made between wavelength calibration and wavelength registration, both of which affect the wavelength uncertainty. The wavelength calibration for a scanning instrument is the relation between the motor steps that determine the grating angle and the monochromator wavelength, and is determined typically from the emission lines of a Hg lamp. The wavelength calibration for a spectrograph is the pixel to wavelength mapping. The wavelength calibration is in general a non-linear function of motor steps or pixels. The two EPA instruments and the NSF_SUV instrument typically use several Hg lines for wavelength calibration and the ASRC_RSS, NIST, USDA_U1K and YES_RSS instruments use several lines from Hg and Cd lamps for the wavelength calibration. Therefore, the lines from the HeCd laser and the Zn lamp are especially valuable for determining the wavelength uncertainty since typically these are not used for the original calibrations of the instruments. For the scanning instruments, the wave length registration is a fixed offset of motor steps, determined from a known position provided by the 302.3 nm line of Hg for the EPA instruments performed after each solar scan, and the 296.7 nm Hg line for the NSF_SUV instrument performed each day at 0500 UTC. During the Intercomparison, the NIST instrument scanned lines of Hg and Cd as a check after each half hour solar scan to see if the instrument error had exceeded its 0.02 nm uncertainty limit, but no adjustment was necessary. The USDA_U1K instrument scanned the 297.6 nm Hg line after each half hour solar scan as a check but no adjustment was made. The ASRC_RSS instrument externally scanned a HgCd lamp each day to determine the pixel to wavelength mapping.

The wavelengths of emission lines from gas lamps are known to a high degree of accuracy. However, the relative intensities of these lines change with lamp and operating condition. An Oriel Model 6035 Hg emission lamp was used because of recent measurements of the relative intensities of the lines from this particular model of lamp [16, 17].

#### 5.2.2 Experimental Procedure

The Hg, Cd, and Zn emission lamps were placed, separately, horizontally and as close as practical over the diffuser of the instrument. The lamps were warmed up for 10 min and the instrument performed a spectral scan. The ASRC_RSS, EPA_101, EPA_114, NIST, NSF_SUV, USDA_U1K and YES_RSS instruments performed spectral scans over their entire operating ranges at 0.08 nm, 0.03 nm, 0.03 nm, 0.04 nm, 0.02 nm, 0.005 nm, and 0.04 nm increments, respectively.

#### 5.2.3 Data Analysis

The bandwidths of the lamp emission lines are calculated as described in Sec. 5.1.2. Baseline subtraction was performed prior to calculation of the bandwidths of the lamp emission lines. This is important for spectral scans of lamp emission lines because of the underlying continuous emission from these lamps. The baseline signal is described by a linear fit of the signals at wavelengths that differ by 1.5 bandwidths from the wavelength of the peak signal. For unresolved multiple lines in emission lamps, the factor is increased from 1.5. to 2.0. The signals and wavelengths for the first five consecutive data points that lie outside this range are averaged and fit with a straight line to yield baseline signal as a function of wavelength. This fit is subtracted from the signals within the range. There is obviously an interplay between the baseline subtraction and the bandwidth, but a consistent bandwidth can be obtained after only one or, at most, two iterations between baseline subtraction and the centroid calculation. Only the bandwidths for single lines were taken to be indicative of the bandwidth of the instrument at that wavelength. The actual centroids of the lines were calculated from the wavelengths and relative intensities of the lines for that particular model of Hg lamp and from the published values for Cd and Zn [18].

#### 5.2.4 Results and Discussion

**Fig. 5.3:**
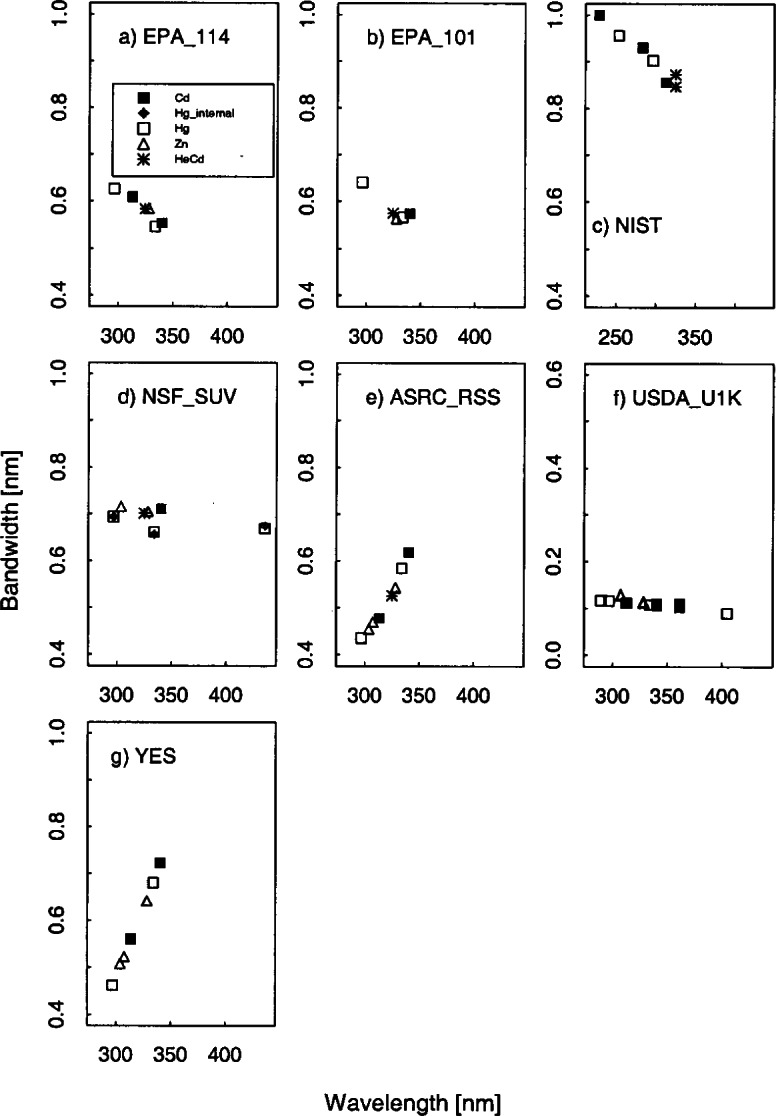
Bandwidth as a function of wavelength for the instruments indicated in each panel from high-resolution spectral scans of the singlet lines from the sources indicated in the legend.

**Fig. 5.4:**
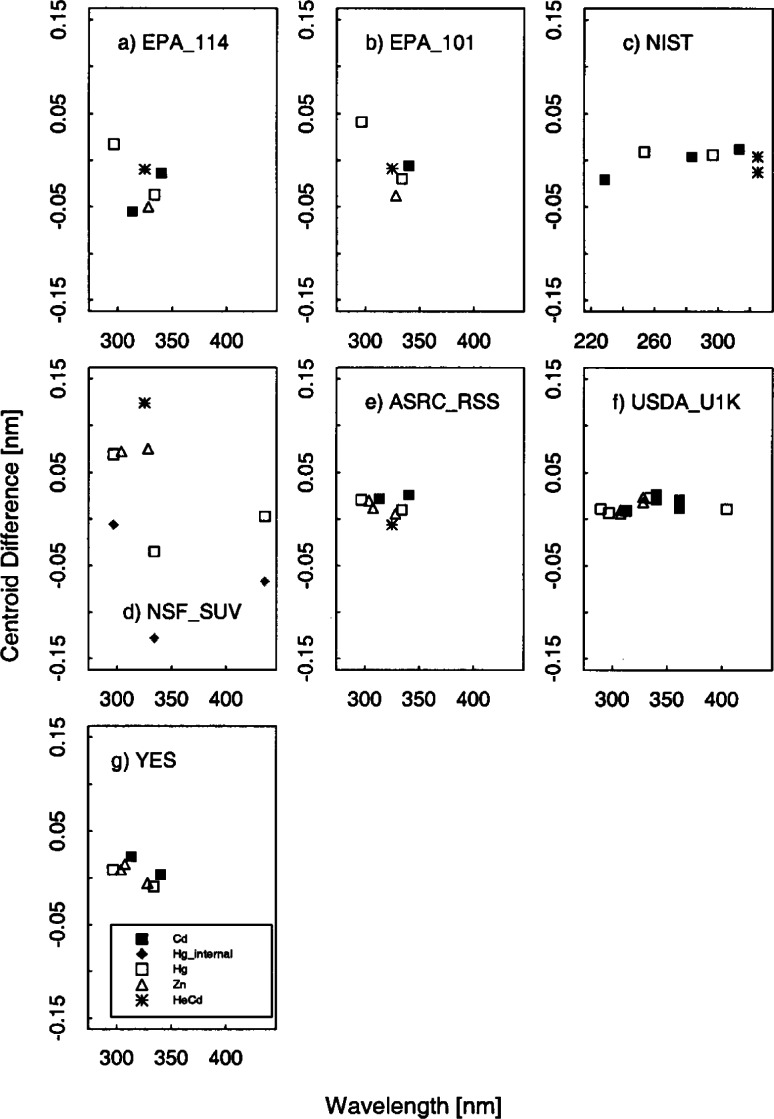
Centroid difference between the calculated and actual values for the instruments indicated in each panel from high-resolution spectral scans of the lines from the sources indicated in the legend, demonstrating the wavelength uncertainty of each instrument.

The bandwidths calculated from the singlet Hg, Cd, and Zn lines and the HeCd line are plotted in Fig. 5.3 as a function of wavelength. The differences between the calculated and actual centroids of the Hg, Cd, Zn, and HeCd lines are plotted in Fig. 5.4 as a function of wavelength.

The nominal band-pass of the two spectrographs, the ASRC_RSS and the YES_RSS instruments, are approximately 0.6 nm which was designed to coincide with the Brewer spectroradiometer and to improve throughput. Note that later versions of the UV_RSS instruments with the 1024 nm × 256 nm CCD array typically are built to achieve 0.3 nm FWHM at 296.7 nm. The bandwidths of the two spectrographs increase with increasing wavelength by 0.82 %/nm and 0.97 %/nm, and this change is consistent between measurements from the different lamps. The FWHM when expressed in pixels is approximately pixel independent, but when converted to units of nanometers is approximately linear with wavelength. The nominal bandwidths of the USDA_U1K and the NSF_SUV are 0.1 nm and 0.7 nm, respectively, and are essentially wavelength independent. The nominal bandpasses of the EPA and NIST instruments are 0.6 nm and 0.8 nm, respectively. The bandpasses of the EPA_101, EPA_114, and NIST instruments decrease with increasing wavelength. The decrease in bandwidth with wavelength for the three instruments is 0.31 %/nm, 0.29 %/nm, and 0.18 %/nm, respectively.

The wavelength uncertainty is determined from the difference between the published values of the centroids for the particular model of Hg lamp [18] and the measured centroids. In general, the wavelength uncertainties were consistent between measurements of the various lamps. The deviations are larger for the NSF_SUV instrument, as explained below. The two Brewer instruments (EPA_101 and EPA_114) appear to have a distinct change in the centroid differences at 325 nm where the filter change occurs. After 325 nm, there is a systematic trend toward decreasing centroid differences with increasing wavelength. Possibly the original wavelength calibration for the Brewer instrument is not representative of the conversion from steps to wavelength and could be improved. In general, for the EPA_101 and EPA_114 instruments, the RMS of the residuals of the centroid differences is 0.029 nm and 0.027 nm, respectively.

The mean centroid offset for the NIST instrument is negligible (0.00014 nm) with a RMS of the residuals of the centroid differences of 0.012 nm, which is less than the 0.02 nm specification. The NSF_SUV instrument wavelength uncertainty results showed significantly larger scatter than expected. The mean of the centroid differences for the NSF_SUV instrument using lines from the Hg, Zn lamps and HeCd laser is −0.06 nm and the RMS of the residuals of the centroid differences is 0.12 nm. The mean centroid offsets for the Cd lines that were determined on a separate day and are not shown in Fig. 5.4 are consistently off by +0.3 nm. These results for the NSF_SUV instrument are not indicative of the normal operation of the instrument and are a result of a programming error in the wavelength calibration routine that has subsequently been corrected. This wavelength uncertainty however did affect the results of the responsivity determination and the synchronized solar scans during the Intercomparison. See section 6.4 for more details.

The mean centroid offset of the ASRC_RSS instrument is +0.013 nm with a RMS of 0.010 nm. The USDA_U1K instrument had a RMS of 0.007 nm with an offset of +0.016 nm. The centroid differences of the YES_RSS instrument had nearly zero offset (+0.007 nm) with a RMS of 0.011 nm.

### 5.3 Spectral Irradiance Responsivity

#### 5.3.1 Introduction

Measuring the spectral irradiance responsivity (hereafter termed simply the responsivity) of the instruments with the NOAA standard lamps was the most important characterization performed at the Intercomparison. At the previous Intercomparisons, the responsivity was determined by the participant and by NIST and NOAA to show the agreement between the two spectral irradiance scales. However, at this Intercomparison the responsivity determined by the participants was not performed. This occurred primarily because many of the participants did not bring their own field calibration systems and previous Intercomparisons showed that its responsivity can change when an instrument is moved, highlighting the need for a field calibrator to perform in-situ responsivity measurements as opposed to laboratory responsivity measurements prior to field placement [4]. The responsivity was determined preferably three times for each instrument to assess the temporal stability of the instruments and to use the most recent responsivity of each instrument for the synchronized solar irradiance measurements.

As stated above, previous Intercomparisons showed that moving the instruments after calibrating caused measurable changes in the responsivity; therefore, this year the instruments were calibrated outdoors on the concrete pads where the instrument remained for the entirety of the Intercomparison. The NOAA standard lamps were operated in the field calibration unit whose performance was demonstrated at the previous Intercomparison [5, 6]. Experimental problems with the YES_RSS instrument had not been resolved during this stage of the Intercomparison and therefore its responsivity was not determined.

#### 5.3.2 Experimental Procedure

Details of the NOAA field calibration unit are given in a separate paper [8]. Briefly, the field calibration unit consists of three circular baffles, 45 cm in diameter and separated by 15 cm, with a mount for a horizontal lamp on the top baffle. A light trap above the lamp and shrouding around the baffles enclose the lamp, isolating it from the surroundings, and the unit mounts on an interface plate, which is the key to the utility of the field calibration unit. Each instrument has an interface plate specifically designed to fit around the diffuser and rest on top of the instrument. The interface plate also sets the distance from the diffuser to the lamp at 50.0 cm by using spacers machined to the appropriate height. The lamp mount on the field calibration unit was adjusted once to center the lamp 50.0 cm above the diffuser.

The spectral irradiance of the 1000 W FEL-type NIST standard lamps, designated 96598 and 96599, had been determined by NOAA in the horizontal position using a method similar to the one described previously [19]. The spectral irradiance of the 1000 W FEL-type NIST standard lamp, designated E-002, had been determined by NIST in the horizontal position also using the method described previously [19]. The responsivity of each instrument was determined with the calibrated lamps mounted horizontally in the field calibration unit.

For all determinations of responsivity using a NIST or NOAA lamp, spectral scans were performed with a 3.5 cm wide shutter halfway between the lamp and the diffuser to measure the diffuse signal, and without the shutter to measure the total signal. For both Brewer instruments, the wavelength registration was set prior to measuring the responsivity. The EPA_101 and EPA_114 instruments performed spectral scans from 286.5 nm to 360 nm at 3.5 nm increments with increasing wavelength for both the diffuse and the total signal. Spectral scans with the NIST instrument were performed from 250 nm to 400 nm in 10 nm increments. Spectral scans with the NSF_SUV instrument were from either 270 nm or 250 nm to 400 nm with a PMT voltage of 800 V. These scans were at a 1.0 nm increment with increasing wavelength with scans for the diffuse and total irradiance. In addition, scans were performed with the internal shutter closed to measure the dark signal. Both the diffuse and total signals from the SERC instrument were collected for nine minutes. The USDA UV-MFRSR instruments (USDA_270, USDA_386, USDA_387) measured diffuse and total signals for 10 minutes. The ASRC_RSS spectrograph collected signals from 295.7 to 349.0 nm at approximately every 0.08 nm for both the diffuse and total signals. The USDA_U1K instrument performed spectral scans from 280 nm to 410 nm at a 1 nm increment with increasing wavelength for the diffuse signal and for the total signal. A schedule of the spectral scans of standard lamps is given in Table 5.2, along with the corresponding instrument temperatures if available.

There were problems associated with using the adaptor plates for the field calibration unit on the ASRC_RSS and USDA_U1K instruments. An incorrect fit of the adaptor plate to each of these instruments resulted in an increased uncertainty in the responsivity measurements due to alignment, as seen in Table 5.3.

#### 5.3.3 Data Analysis

From spectral scans of a standard lamp, the responsivity is given by dividing the signal by the lamp irradiance. For the NIST and NOAA standard lamps, the signal was the direct signal, given by the difference between the total signal and the diffuse signal where the diffuse signal was determined by placing a shutter in front of the lamp during the irradiance measurement. Typically, the responsivity determined by the participants for use in their networks do not use a shutter to determine the diffuse signal and the signal is therefore the total signal. The spectral irradiance of the standard lamps was fit with a cubic spline interpolation to the wavelengths of the signals. The NOAA standard lamps had been calibrated from 250 nm to 400 nm, which covers the wavelength range measured during this Intercomparison.

The uncertainty analysis for the responsivities is similar to the approach given in previous Intercomparisons [4, 6], where the details are presented in Appendix D of Ref. [5] and the specifics for each instrument are given in Appendix B here. Components of uncertainty arise from the standard lamp (spectral irradiance, size of diffuser, goniometric distribution, and current), the alignment of the lamp, and the instrument (wavelength and signal). The relative standard uncertainties arising from each component for both random and systematic uncertainties are given in Table 5.3 at selected wavelengths for the first determination of responsivity with the field calibration unit. The relative standard uncertainties are combined in quadrature for both random and systematic effects. The relative standard uncertainty in the relative difference between two responsivities determined by the NOAA standard lamp includes components of uncertainty arising only from random effects. The greatest systematic component is the irradiance of the standard lamp, while the greatest random component is the signal. Note that for several of the prototype instruments, the uncertainty arising from the alignment of the field calibrator over the instrument can also have a significant contribution to the overall measurement. This occurred for instruments where the plate that adapted the field calibrator over the instrument’s diffuser did not fit properly as described above.

#### 5.3.4 Results and Discussion

**Fig. 5.5:**
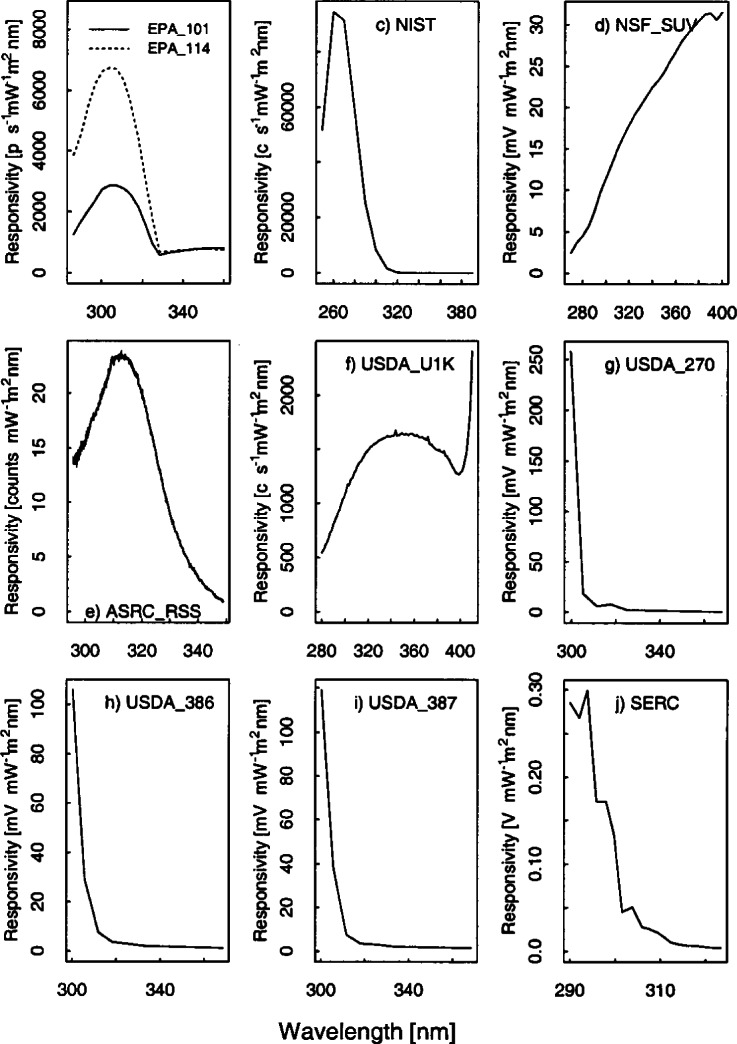
Responsivity as a function of wavelength for each instrument indicated in the panels.

The responsivities of the instruments as a function of wavelength are shown in Fig. 5.5. The peaks in the responsivities of the EPA instruments between 300 nm and 320 nm are primarily due to the shape of the spectral response of the NiSO_4_ filters. The abrupt change in responsivity at 325 nm is due to a change from a NiSO_4_/UG-11 filter combination with the grating operating in second order for wavelengths at and shorter than 325 nm to a UG-11 filter with the grating also operating in second order for wavelengths longer than 325 nm. The responsivity of the ASRC_RSS, EPA_101, EPA_114, NIST, and SERC instruments are designed to peak at the shorter wavelengths where the solar irradiance signal is low and decrease at larger wavelengths where the solar irradiance signal is larger. The shape of the responsivity for the ASRC_RSS is largely controlled by the detector array sensitivity and the fore-optics throughput, the latter combining the solar blind filter transmittance and the transmittance of the fused silica lens. The responsivity of the NIST, NSF_SUV, and USDA_U1K instruments are dominated by the fore-optics, monochromators, and PMT. The responsivity of the SERC instrument is dominated by the PMT.

**Fig. 5.6:**
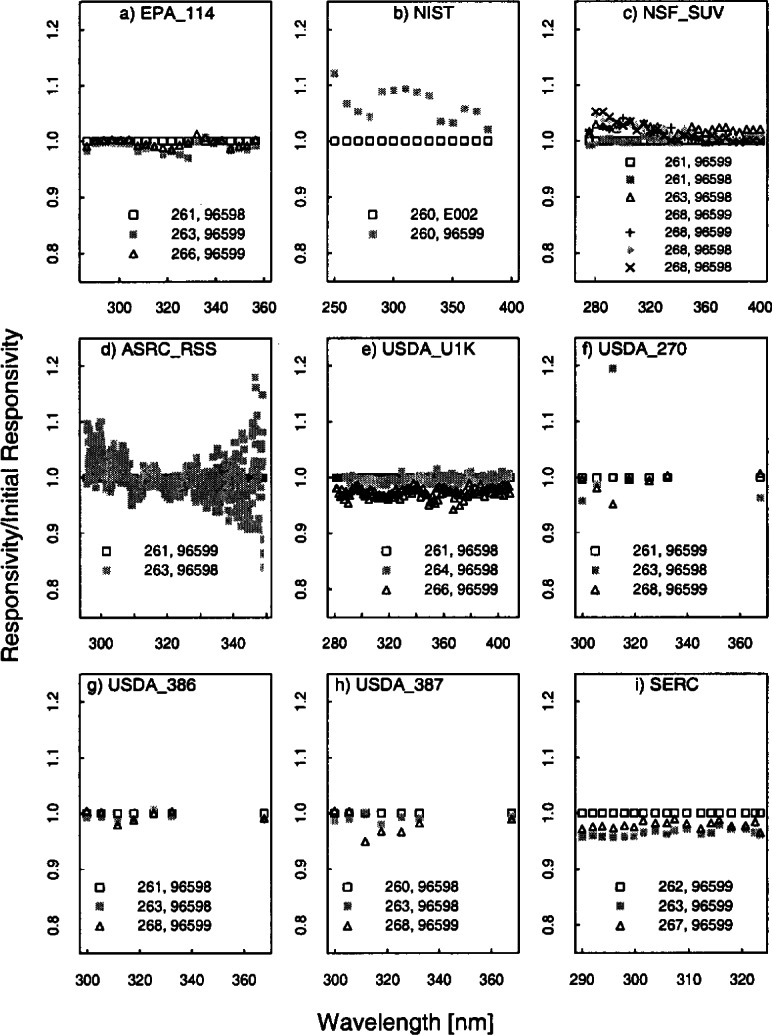
The ratio of the responsivity on the indicated day to the responsivity determined on the first day, using the NIST or NOAA standard lamps, as a function of wavelength, indicating the variation of the responsivity with time and lamp. The instruments are indicated in each panel, the Julian day on which the responsivities were determined and the NIST or NOAA lamps used are indicated in the legends.

**Fig. 5.7:**
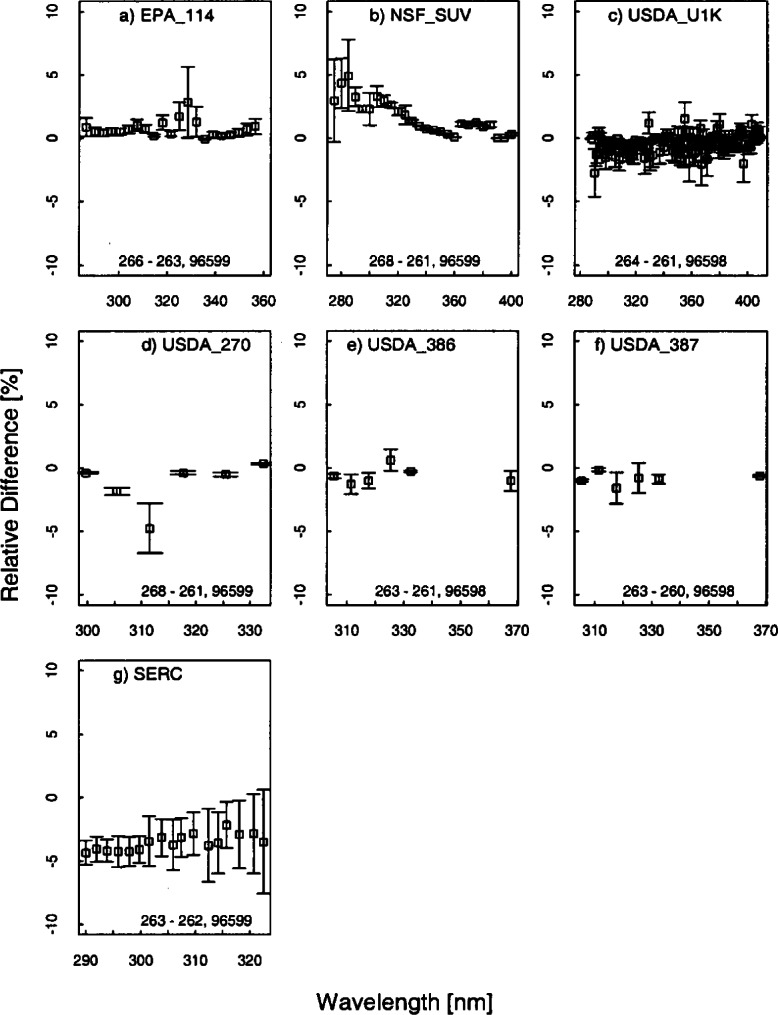
The percent relative difference of the responsivity determined on two different Julian days, indicated in the legend, with the same lamp, also indicated in the legend. This figure demonstrates the temporal stability of the instrument.

Figure 5.5 gives all of the responsivities determined with the three NIST and NOAA lamps (NOAA-#96598, NOAA-#96599, and NIST-#E002). The same data as in Fig. 5.5 is shown in Fig. 5.6 as the ratio of the responsivity on the day given in the legend to the initially determined responsivity using the NOAA and NIST standard lamps for each of the instruments. The temporal stability of the instrument is indicated in the difference between responsivities determined with the same lamp at two different times. The relative difference between the responsivity and the responsivity determined with the same lamp at a later date is given in Fig. 5.7. The vertical bars in Fig. 5.7 are the combined standard uncertainties of the differences using components arising from only random effects. The relative standard uncertainties from random and systematic effects are given in Table 5.3. It is valuable to differentiate systematic from random effects here because the uncertainties from random effects in the responsivity become systematic effects in the uncertainty of the solar irradiance. All of the uncertainty components used a Type B evaluation except the instrument signal, which used a Type A evaluation. The total uncertainties in the responsivities when propagated to the solar irradiance contribute to the uncertainties in the solar irradiance measurements.

For the EPA_114 instrument, the responsivity was fairly stable and decreased by 2.7 % or less over the measured wavelength range from day 263 to day 266 with a mean decrease of 0.7 %. This stability is better than expected because the optics and the detector of the Brewer Mark IV instrument are not temperature stabilized and as a result the responsivity of the instrument is temperature dependent. Temperature measurements during the responsivity determination are given in Table 5.2. For the NIST instrument, the responsivities determined by the two lamps (Fig. 5.6) differ by as much as 12 % at two different times on day 260. This result is primarily caused by electronic problems that began during the Intercomparison and was difficult to resolve outdoors in the rain.

**Fig. 5.8:**
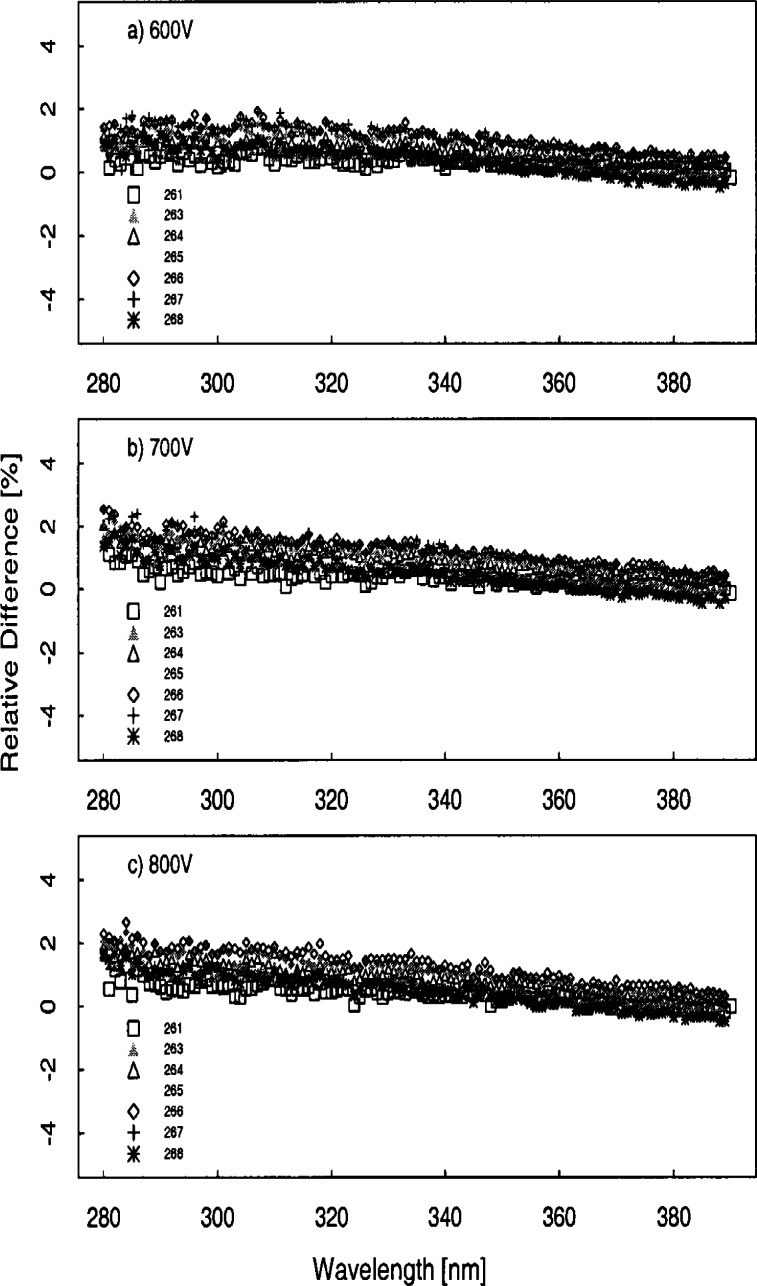
The percent relative difference between signals measured from the internal 45 W lamp of the NSF_SUV instrument at different times and PMT voltages, indicated in each panel from the responsivity determined on Julian day 259, demonstrating the temporal stability of this lamp. The legend indicates the Julian date of the responsivity measurement.

The responsivity of the NSF_SUV instrument determined on day 261 with NOAA lamp #96599 differed by less than 0.7 % from the responsivity determined on day 261 with NOAA lamp #96598 with an average decrease of −0.17 % from the #96599 scan to the #96598 scan. The responsivities determined on day 263 and day 268 are very consistent with each other and agree within 1.8 % regardless of which lamp is used. There is a distinct increase in the responsivity from day 261 to the responsivities determined after this day of up to 5 % at 280 nm. This change is most likely due to a combination of an error in a wavelength calibration program, which will be discussed further in Sec. 6.4, and a drift in the instrument. The internal lamp scans show a drift in the instrument of not more than 2 % over 9 days and is illustrated in Fig. 5.8, which gives the relative difference in responsivity determined with the internal lamp with each succeeding day from the responsivity determined on day 259. Disregarding the wavelength programming error, the NSF_SUV instrument shows very good temporal stability.

The responsivity of the ASRC_RSS instrument shows significant scatter (+/−10 %) from one determination to the next but negligible offset (Fig. 5.6). The relatively large scatter of data points can be explained by low sensitivity of the MOS detector array and low lamp irradiance below 310 nm, and then low signal above 330 nm due to the intentionally low throughput of the fore-optics, as shown in Figure 5.5. Consequently, the relative standard uncertainty of the responsivity measurement at 295 nm is 5.2 % and at 348.8 nm is 14.8 %, which is consistent with Figure 5.6. The responsivity of the USDA_U1K instrument determined using lamp #96598 decreased by a mean of 0.4 % from Julian day 261 to 264 as shown in Figure 5.7 and is within 1.8 % for all but two points. As illustrated in Fig. 5.6 the responsivity determined on day 266 decreased by a mean of 2.6 % from day 261 but used a different lamp. The largest sources of uncertainties in the USDA_U1K signal are the random component from the signal and the uncertainty in the positioning of the standard NOAA lamp over the instrument. The uncertainty components are given in Table 5.3.

There were three UV-MFRSR instruments at the Intercomparison with serial numbers #270, #386, and #387 and the temporal stability of all three instruments were similar. The three instruments showed an approximate mean decrease of 1.2 %, 0.6 %, and 0.9 % over 7 d, 2 d, and 3 d (Fig. 5.7), respectively. The 311 nm channel of the USDA_270 showed a decrease of 5 % over the 8 days that was unexplained. This instrument shows pronounced deviations from day 261 to day 263 (Fig. 5.6) but this was attributed to insufficient warm-up time of the head and was not an indication of the instrument’s stability. The typical warm-up time of the head of an UV-MFRSR prior to measurement of the responsivity should be at least 20 min. Excluding the responsivity determined with an insufficient warm-up time, all three instruments showed changes in the responsivity within 5 %. The responsivity of the SERC instrument decreased by less than 4.4 % over the wavelength range and had a mean decrease in the responsivity of 3.5 % from day 262 to day 263 which is consistent with previous Intercomparisons. The responsivity of the SERC instrument decreased by less than 2.9 % from day 261 to day 267.

The conclusions to be drawn from the determinations of responsivity are similar to those from the previous Intercomparisons. The responsivity changes were within ±5 % indicating relatively good temporal stability. Unlike the results in 1994, the temperature changes between determinations of responsivity for the EPA instruments were not sufficient to illustrate the effect of temperature on responsivity. The responsivities determined outdoors using the NOAA standard lamp were used to calculate the irradiance from the synchronized solar scans. Using a common standard for responsivity simplifies intercomparisons between measured irradiance since differences between spectral irradiance scales are removed from the analysis. Therefore, actual instrument performances can be evaluated more readily. Whether the optics or the detector of the instruments are temperature stabilized is indicated in Table 3.1.

## 6. Solar Irradiance

### 6.1 Introduction

The major goal of the Intercomparison was to have all the instruments measure the solar ultraviolet irradiance concurrently, which was achieved over several days of the Intercomparison. The solar ultraviolet irradiance *E*(_0_) was calculated from the measured signals *S*(*λ*_0_) using the simplified measurement equation given by

$$E(\lambda_{0})=S(\lambda_{0})/R(\lambda_{0}),$$ (6.1)

where *R*(*λ*_0_) is the responsivity for each instrument. This was done to provide a common irradiance scale for all the instruments, thereby removing discrepancies caused by different scales and facilitating comparisons between instruments. Several instruments were having operational problems during the Intercomparison and the unfavorable weather conditions made diagnosing and fixing the problems a daunting task. These instruments included the YES UV-RSS spectrograph, which was having operational difficulties from the start of the Intercomparison, and the NIST instrument, which began to have electronic problems at a later stage of the Intercomparison. Neither of these instruments participated in the synchronized solar scans.

### 6.2 Experimental Procedure

Synchronized spectral measurements of the solar ultraviolet irradiance began on the hour and half-hour from wavelengths of 290 nm to 348 nm at increments of 0.2 nm with 3 s between each wavelength. This range was common to all the instruments; the two EPA instruments, the NSF_SUV, and USDA_U1K instruments extended their scans to 363 nm, 400 nm, and 360 nm, respectively. The clock for each instrument was set daily from a common clock synchronized with the satellite Global Positioning System. The scanning time for the synchronized scans from 290 nm to 360 nm was 17.5 min. The ASRC_RSS instrument is a spectrograph and therefore measures all wavelengths at once. To compare the ASRC_RSS to other scanning spectroradiometers, the wavelength measured at the particular scan time was chosen for synchronized comparisons, as explained in the instrument description section. Other measurements, such as wavelength calibrations and total column ozone, were performed by the two EPA Brewer instruments during the times between synchronized scans. The days, times, and participating instruments for the synchronized solar scans used in the analyses below are listed in Table 6.1. As stated above, both the YES_RSS instrument and the NIST instrument were not operational during the synchronized scans. The EPA_101 instrument was not operating correctly until after 15.0 h UTC on day 267 due to a power supply problem. The EPA_114 instrument had a jammed filter wheel until the morning of day 265, eliminating the earlier data from the analysis.

### 6.3 Data Analysis

For all instruments, the measured signal was corrected before the irradiance was calculated. For the two EPA instruments, the signal was converted to a photon rate [4] with dark subtraction and dead-time correction. Dark subtraction was performed on the NSF_SUV instrument by averaging all the signals at wavelengths shorter than 290 nm and subtracting this value from all the signals of the scan. Dark subtraction and averaging the signals over the 17.5 min of the synchronized scans was performed for the SERC instrument. The average of the dark signals obtained immediately before and after a synchronized scan of the USDA_U1K instrument was subtracted from all the signals of the scan. For the ASRC_RSS instrument, the exposure time of the CCD array was set for 10 s with the shutter open to measure the total irradiance signal, and then the exposure was set for 10 s while the shutter was closed to measure the dark signal. The dark signal was then subtracted from the total signal.

The stray-light rejection of the instruments, shown in Fig. 5.2, can result in relatively large signals at the shortest wavelengths. To account for this, stray-light subtraction was employed for the EPA instruments. The signals at wavelengths shorter than 292 nm were averaged and subtracted from all signals from the scan. It was these signals with the stray-light subtraction that were divided by the responsivity to obtain the solar ultraviolet irradiance. The subtraction used for the NSF_SUV instrument would also correct for stray-light if it exists. The stray-light rejection of the USDA_U1K instrument was sufficient and no correction to the signals at the shortest wavelengths was necessary.

The method used to determine the responsivity of the NSF_SUV instrument during solar scans complicated the data analysis. The usual procedure with this instrument is to transfer the spectral irradiance scale of the external 200 W lamp to the internal 45 W lamp from spectral scans of both lamps with the same high voltage on the PMT. Different high voltages are used for scans of the solar irradiance, and the responsivity of the instrument is dependent upon the high voltage. Therefore, the internal lamp is scanned at least daily at all PMT high voltages that are used for solar measurements. To use the NIST irradiance scale with this procedure, the scale was transferred to the NSF external 200 W lamp from the scan of the NIST standard lamp at 800 V, and this new scale for the external lamp was then used with scans of the internal 45 W lamp. The responsivity of the instrument at any high voltage was determined from the scan of the 45 W lamp at the same high voltage that occurred closest in time to the scan of the solar irradiance. To maintain consistency with the NSF_SUV procedure for responsivity, the responsivities used to calculate the solar irradiance were those determined closest in time to the synchronized scans.

The responsivities of the EPA instruments were determined every 3.5 nm while the solar irradiance scans are determined every 0.2 nm. Because of the filter change out after 325 nm, the responsivities were extrapolated from 328.5 nm to 325.01 nm and from 360 nm to 363 nm using second-order polynomials. The extrapolation for the responsivity of the EPA instrument does cause some additional uncertainties around 325.0 nm for this analysis that are then propagated in the solar irradiance scans. Typically, the EPA Network determines the responsivity at the same wavelengths as the solar irradiance scans and this is therefore not a problem. From Eq. (6.1), the irradiance at a given wavelength is the signal at that wavelength divided by the responsivity at that same wavelength; because the responsivities of the EPA instrument were not determined at all the wavelengths of the synchronized solar scans, the responsivities at these wavelengths were calculated from natural cubic spline interpolations. The days and times of the responsivities for the solar irradiance used by all participating instruments on day 267 are given in Table 6.2.

### 6.4 Results and Discussion

**Fig. 6.1:**
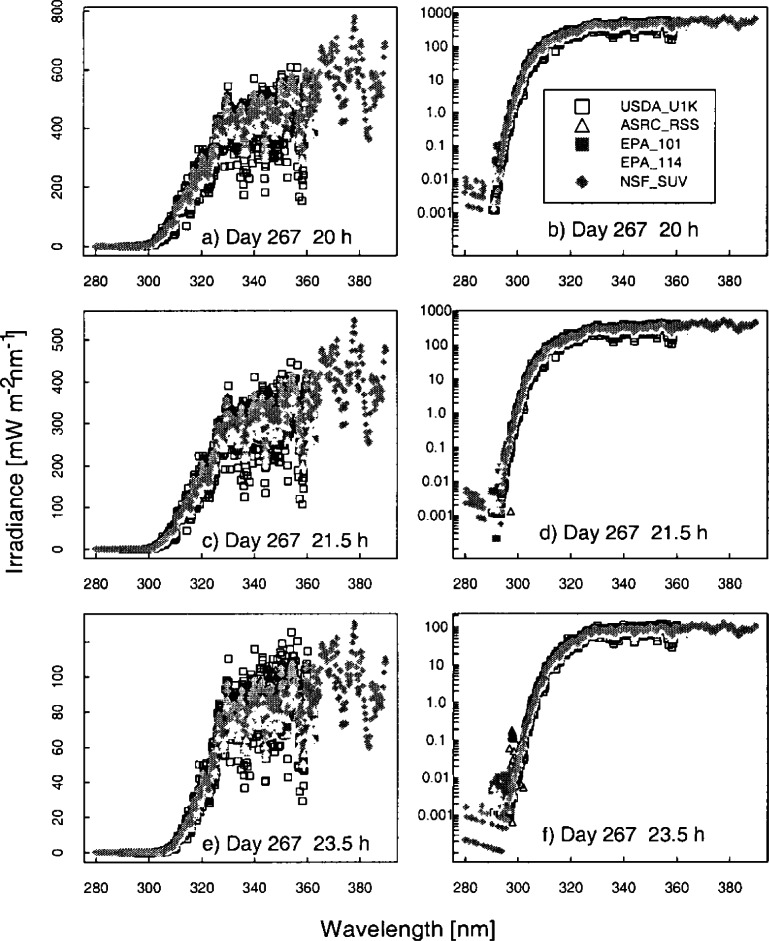
Solar irradiance on a (a,c,e) linear and (b,d,f) logarithmic scale as a function of wavelength determined by the instruments indicated in the legend on day 267 at 20.0 h, 21.5 h, and 23.5 h UTC.

The solar irradiance as a function of wavelength determined by the scanning spectroradiometers and spectrograph instruments from a synchronized spectral scan on day 267 at 20 h, 21.5 h, and 23.5 h UTC is shown in Fig. 6.1. Day 267 is chosen for the remainder of the comparisons because it is the only day that most instruments were operational and was also a clear sky day. The irradiance is plotted on a linear scale in Fig. 6.1(a,c,e) and on a logarithmic scale in Fig. 6.1(b,d,f). This figure illustrates the challenges encountered in accurately measuring the solar ultraviolet irradiance, especially in the UV-B wavelength region, and of comparing the results between instruments. The outstanding feature of ground-level solar ultraviolet irradiance is its rapid decrease with decreasing wavelength in the UV-B region due to absorption by ozone, as illustrated in Fig. 6.1(b,d,f). The irradiance decreases by five to six orders of magnitude from 325 nm to 290 nm, which imposes stringent requirements on the instruments in terms of wavelength accuracy and stray-light rejection (see Sec. 5). In the region of steepest decrease, a relatively small uncertainty in wavelength translates into a large uncertainty in irradiance. An accurate measurement of the irradiance at the shortest wavelengths requires the best possible stray-light rejection so the signal is not dominated by light from wavelengths longer than the nominal one.

The moderately structured nature of the solar spectral irradiance, as shown in Fig. 6.1(a,c,e) for wavelengths greater than 310 nm, complicates comparisons between instruments. While the structure of the spectral irradiance is consistent among instruments, with maxima and minima occurring at approximately the same wavelengths, the effect of the different bandwidths is also apparent. As the bandwidths of the instruments increase from USDA_U1K, ASRC_RSS, EPA, to NSF_SUV the measured spectral irradiance becomes smoother. The maxima and minima measured by the USDA_U1K instrument are more pronounced than those measured by the EPA instruments, for instance, and virtually no structure is evident with the filter instruments (SERC, UVMFRSR). The solar irradiance plotted on a logarithmic scale gives an indicator of which instruments are capable of measuring below 300 nm.

The problem remains of how to compare the solar irradiance measured by instruments with different bandwidths. While deconvolution and spectral synthesis techniques are being investigated, for convenience the approach taken for this paper is to convolve the irradiance with a common slit-scattering function [20, 21]. This assumes the instruments are accurately measuring the solar irradiance, so that the convolution is approximating the solar irradiance that would be obtained by a hypothetical instrument with a given slit-scattering function. The results are presented in order of increasing complexity of the slit-scattering function used in the convolution. In the simplest case, the solar irradiance from the scanning spectroradiometers and spectrograph instruments (ASRC_RSS, EPA_101, EPA_114, NSF_SUV, and USDA_U1K) are compared by convolving each irradiance with a 1 nm FWHM ideal triangular slit-scattering function. Therefore, the effect of this convolution is that all the instruments have the same 1 nm triangular bandwidth. This method is used to compare all the instruments except the narrow band filter instruments. The final convolution technique allows comparisons among all the instruments. The goal is to produce a pseudo-measurement from the spectral data that approximates what the filter instrument would see by using the filter transmittances of the SERC, USDA_270, USDA_386 and USDA_387 instruments. This approach does not require any additional knowledge about the atmosphere, solar spectral irradiance, or radiative transfer. The irradiance *E_j_* at filter channel *j* for each scanning instrument is given by

$$E_{j}=\sum_{i}E(\lambda_{i})\tau_{j}(\lambda_{i})/\sum_{i}\tau_{j}(\lambda_{i}),$$ (6.2)

where *i* indexes the wavelengths λ*_i_* and *τ_j_* is the filter transmittance for channel *j*.

The relative standard deviation is the quantity used to quantify the agreement between instruments and is the standard deviation of the solar irradiance divided by the average irradiance at each wavelength. The relative difference is also used to convey the spread of values and is a good indicator of each instrument’s performance. The relative difference is given by the solar irradiance measured by a particular instrument minus the average of the solar irradiance divided by the average solar irradiance at each wavelength. Because all the instruments performed synchronized spectral scans under nominally identical conditions, they are assumed to have been exposed to the same spectral irradiance. However, each instrument measured an independent value for the solar irradiance, and therefore the relative difference of these independent values is used to indicate the agreement between instruments.

**Fig. 6.2:**
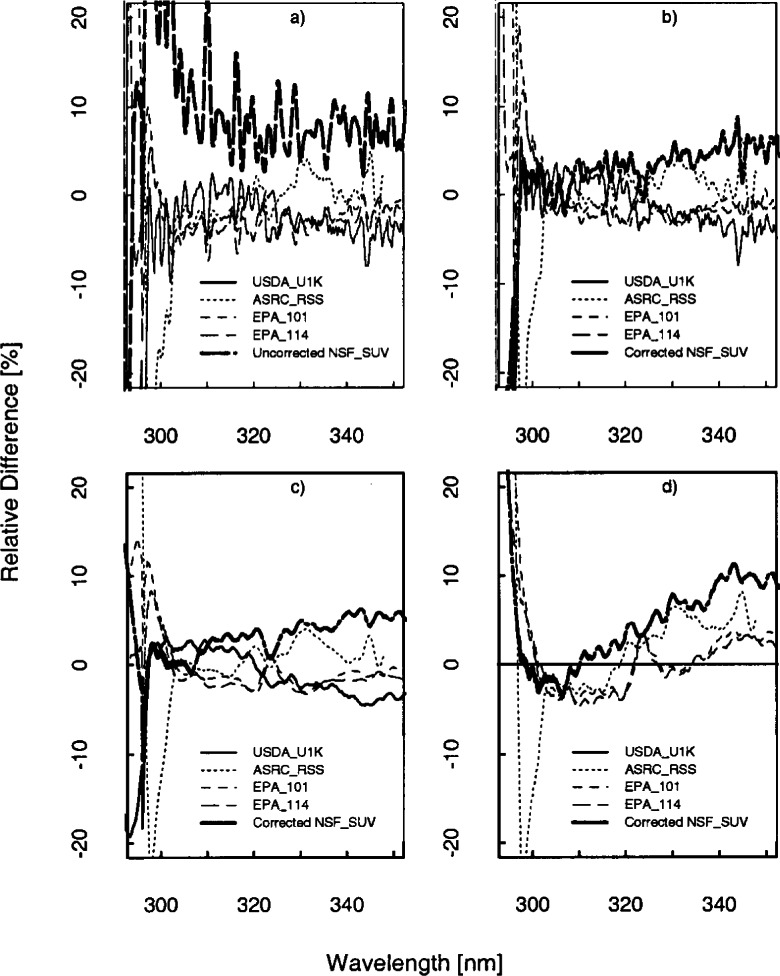
a) and b) The relative differences between the solar irradiance convolved with a 1 nm triangular slit-scattering function as a function of wavelength for the instruments indicated in the legend and the average solar irradiance on Julian day 267 at 16.5 h UTC. c) The relative differences between the solar irradiance convolved with a Gaussian slit-scattering function as a function of wavelength for the instruments indicated in the legend with the average solar irradiance on day 267 at 16.5 h UTC. d) The relative differences between the solar irradiance convolved with a Gaussian slit-scattering function as a function of wavelength for the instruments indicated in the legend with the solar irradiance from the USDA_U1K instrument on Julian day 267 at 16.5 h UTC.

The results presented here focus on the irradiance measured on day 267 at 16.5 h UTC since all the instruments were operating correctly and the sky was clear. The relative difference of the solar irradiance convolved with a 1 nm triangular slit-function for the instrument given in the legend to the average solar irradiance of all the instruments given in the legend is shown in Fig. 6.2 a–c. The relative differences in Fig. 6.2 a use the triangular convolution and range from −8 % to 25 % for wavelengths greater than 305 nm. This is greater than expected from the propagation of uncertainties. The NSF_SUV instrument is noticeably higher than the other spectroradiometers and also shows significant spectral structure (Fig. 6.2 a). The significant spectral structure for the NSF_SUV data in Fig. 6.2 a suggests a wavelength calibration problem. A spectral shift of the data of approximately 0.3 nm corrects the discrepancy. A further analysis revealed a programming error in a wavelength calibration routine used by the NSF_SUV instrument. This program is not generally used in routine remote operations but was used as an additional check. The program is designed to determine the position of the 253.652 nm Hg line in measured wavelength scans, subtract this position from the nominal value of 253.652 nm and adjust the wavelength calibration of the instrument by this difference. Unfortunately, the measured position was stored as an integer, i.e., 254 nm. During several occasions, including day 267, the wavelength calibration was incorrectly adjusted by the difference of 254 and 253.652, which resulted in a wavelength error of 0.348 nm for day 267. The corrected NSF_SUV solar irradiance data are plotted in Fig. 6.2 b and and show a significant improvement in the agreement with the other instruments. The wavelength calibration was also checked and recomputed for the responsivity measurements. With the corrected NSF_SUV solar irradiance data, the spectral structure has been reduced and the overall agreement with the other instruments has increased. In Fig. 6.2 b, the relative difference has decreased to a range from ±6 % for wavelengths greater than 305 nm using the corrected NSF_SUV solar irradiance data.

In contrast, convolving with a Gaussian slit-scattering function results in relative differences that range from ±5 % and are approximately the same as those expected from the propagation of uncertainties. Here, the minima and maxima have decreased from the triangular convolution in Fig. 6.2 b. The decreased relative differences relative to those obtained with a triangular convolution are primarily a result of convolving with a function which includes all the wavelengths from the spectral scan, and not a limited number as with the triangular convolution. However, convolving with a Gaussian function is a reasonable method for comparing irradiance measured by instruments with different bandwidths since all the convolved irradiances include the bandwidths of all the instruments. The discrepancies between instruments increase significantly below 300 nm and are in part due to differences in stray-light rejection (see Sec. 5.1). An instrument with poor stray-light rejection is expected to have a larger signal than the true value. Because the USDA_U1K instrument was shown to have the best stray-light rejection (≈10^−10^), the percent relative difference is plotted relative to the USDA_U1K solar irradiance data (Fig. 6.2 d). The ASRC_RSS instrument is showing unusual behavior starting around 303 nm, becoming more pronounced as the day progressed; this feature is unexplained. Excluding the data from the ASRC_RSS instrument, the USDA_U1K instrument has the lowest irradiance at the shorter wavelengths and the NSF_SUV instrument, which is also a double monochromator, measures less than the Brewer spectroradiometers at these shorter wavelengths.

**Fig. 6.3:**
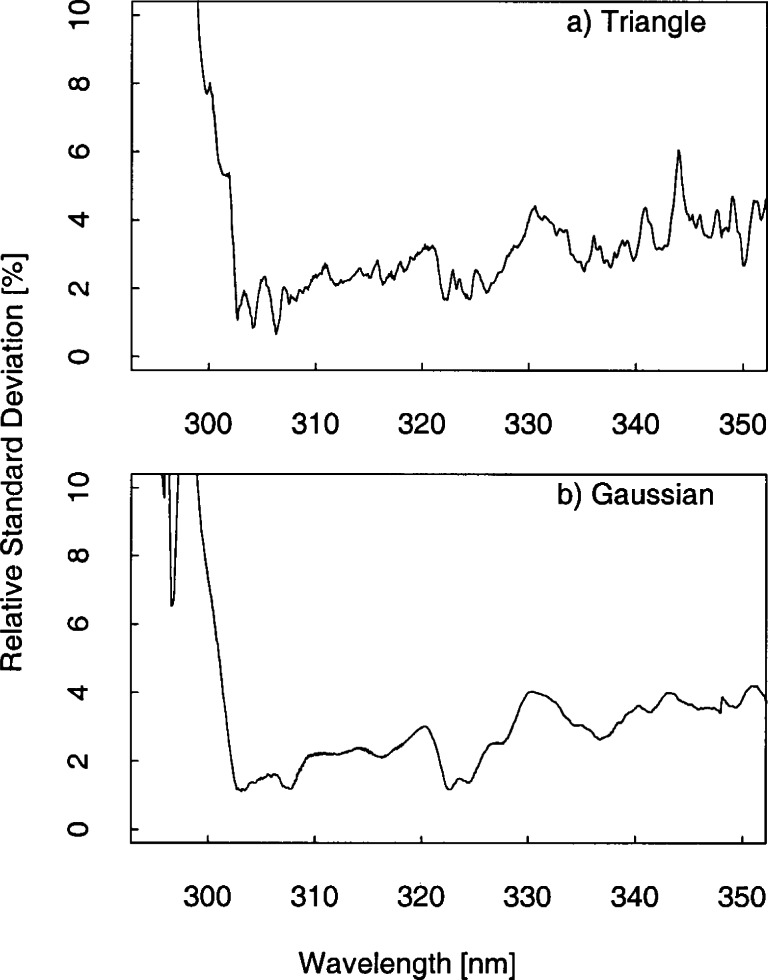
The relative standard deviation of the solar irradiance at 16.5 h UTC of the spectral instruments convolved with a (a) triangle slit-scattering function and a (b) Gaussian slit-scattering function.

The relative standard deviations of the solar irradiances measured by the five instruments from Figs. 6.2 b and Figs. 6.2 c are shown in Fig. 6.3. For the triangle slit scattering function the relative standard deviation is less than 5 % for wavelengths longer than 305 nm (Fig. 6.3 a) and for the Gaussian slit scattering function the relative standard deviation is less than 4 % (Fig. 6.3 b). In general, the convolution removes most of the discrepancies due to differences in bandwidth. The differences in bandwidth and the wavelength uncertainties among the instruments are responsible for much of the spectral structure observed in the relative differences. Using the same analysis used previously [4] and assuming a 2 % relative standard deviation from all other sources, a wavelength uncertainty of approximately 0.1 nm would account for the relative differences in Fig. 6.2 d.

**Fig. 6.4:**
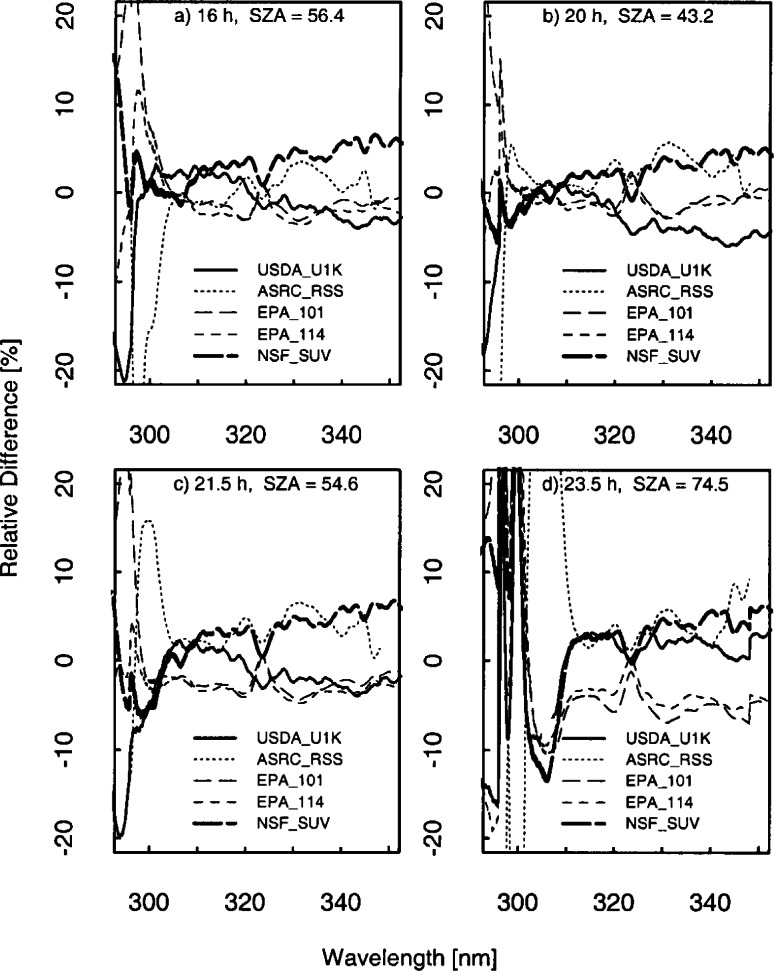
(a) (d) The relative differences between the solar irradiance convolved with a Gaussian slit-scattering function of the instrument indicated in the legend to the average convolved solar irradiance as a function of wavelength on day 267 at 16 h, 20 h, 21.5 h, and 23.5 h UTC (56.4°, 43.2°, 54°, and 74.5° SZA, respectively).

Figure 6.4 illustrates the relative differences between instruments with changing solar zenith angle (SZA). Figure 6.4 gives the solar irradiance convolved with Gaussian slit-scattering function versus wavelength for the instruments indicated in the legend for four different times indicated in the title (16 h, 20 h, 21.5 h, and 23.5 h UTC or 56.4°, 43.2°, 54°, and 74.5° SZA). The relative differences generally increase with increasing solar zenith angle, partly due to the increased uncertainties in the measurements, but also due to differences between the instruments. This could possibly be due to either the differences in the angular response of the detectors or the linearity of the detectors. Interestingly, the EPA instruments are grouped together and decrease relative to the USDA_U1K, ASRC_RSS, and the NSF_SUV as the solar zenith angle increases. This is consistent with their angular response because the angular response of the USDA_U1K, ASRC_RSS and the NSF_SUV instrument are in general closer to ideal than the two EPA instruments. Instruments with a non-ideal angular response measure less irradiance than the true irradiance and as the solar zenith angle increases the angular response error typically increases. For instance, the Brewer instruments are estimated to measure approximately 3 % low at high sun and 8 % low at low sun due to angular response error based on a typical measured angular response and model calculations of the sky radiance on a clear sky day, but of course these estimates depend on the specific sky conditions [22]. The angular responses of all the instruments were not available at the time of this analysis, but it would be useful in the future to apply the angular corrections to the solar irradiance data of each instrument and observe if this improves the agreement between irradiance measured by the instruments. As the day progresses, the ASRC_RSS instrument is measuring significantly more than the other instruments at the shorter wavelengths and is outside the uncertainty limits. It is unclear what is causing this because the effect is not seen at similar zenith angles earlier in the day. It is usually instructive to compare the irradiance measured by each instrument on different days. This is usually done to assess how the different instruments respond to changing ozone conditions and changing cloud cover relative to one another. However, because of poor weather, instrumental difficulties, and a short-term power outage, there were no other days where all the instruments were operating to do this comparison.

**Fig. 6.5:**
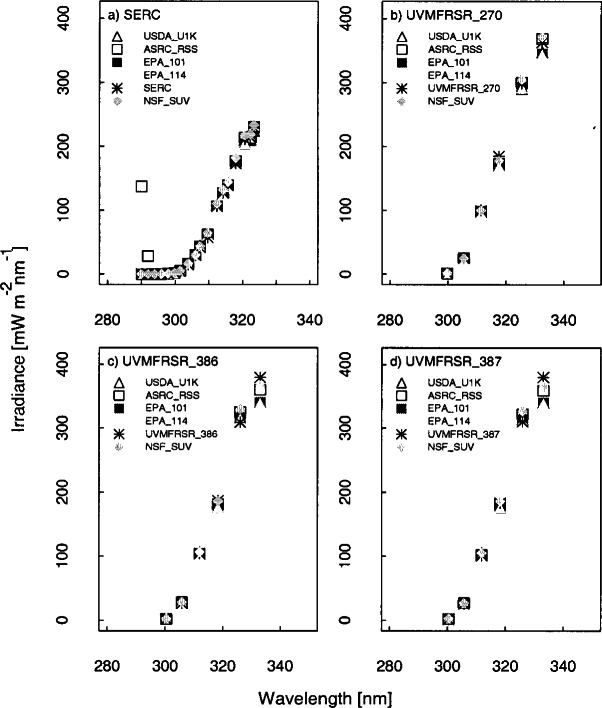
Solar irradiance as a function of wavelength of the filter instrument (a) SERC, (b) USDA_270, (c) USDA_386, and (d) USDA_387 and the solar irradiance of the scanning spectoradiometers and spectrographs indicated in the legend convolved with the filter functions of the filter instruments indicated in the title.

**Fig. 6.6:**
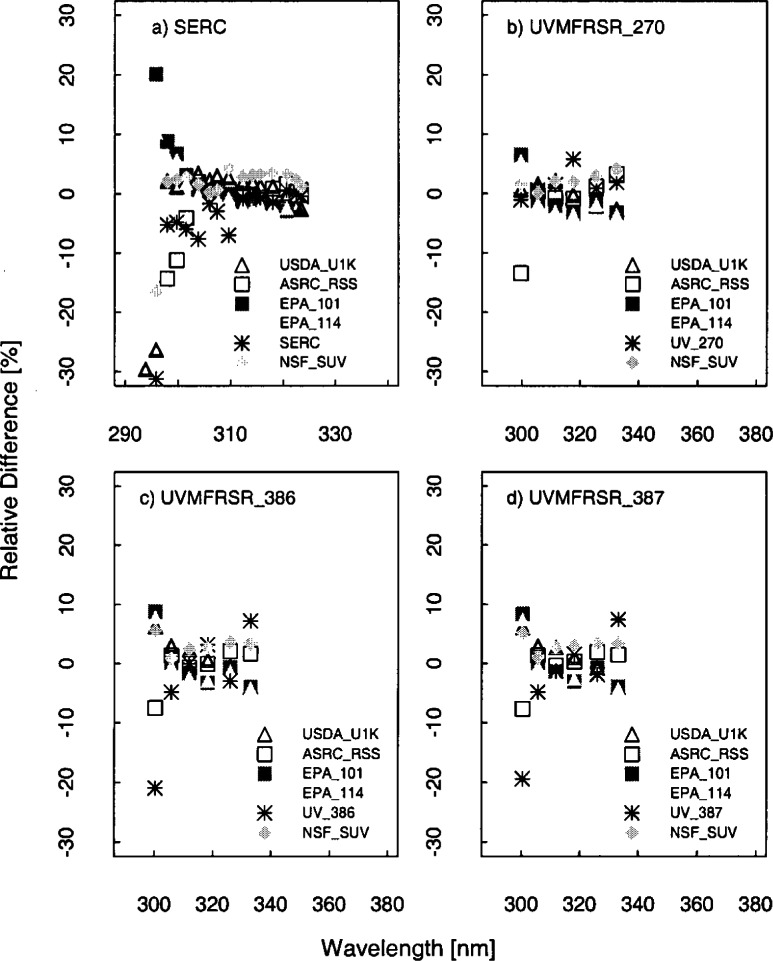
Relative difference as a function of wavelength of the solar irradiances measured by the instruments indicated in the legend to the average solar irradiance on day 267 at 16.5 h convolved with the slit-scattering functions of the filter instruments indicated in the title.

In the final comparison, the solar irradiance of the scanning spectroradiometers and spectrographs are convolved with the filter transmittances of the SERC and each of the three UVMFRSR’s, and are compared to the solar irradiance measurements from each of the filter instruments (SERC, UVMFRSR_270, UVMFRSR_386, UVMFRSR_387). Figure 6.5 plots the solar irradiance as a function of wavelength for day 267 h and 16.5 h UTC for the four filter instruments and Fig. 6.6 plots the relative difference to the average. The important point in these figures is how the solar irradiance from the filter instrument (the stars) compares to the convolved spectroradiometer data. Figure 6.6 a compares the solar irradiance from each of the spectroradiometers convolved with the SERC filter transmittances and the SERC solar irradiance data. The SERC instrument agrees within 8 % to the filter-weighted spectral irradiance data for all filters except the four shortest filter wavelengths (<296 nm nominal wavelength). Certainly, many of the spectral instruments have difficulty measuring the shorter UV wavelengths and comparing to the average at these wavelengths is not too informative. The SERC channels 294 nm and 296 nm agree within 10 % to the USDA_U1K instrument; however, the two shortest wavelength SERC channels (not shown) are over 90 % higher than the USDA_U1K which has the best stray-light rejection. The comparisons at other solar zenith angles are similar. Figure 6.6 b–d compares the solar irradiance from the spectral instruments convolved with each of the UVMFRSR filter functions (USDA_270, USDA_386, USDA_387) for day 267 at 16.5 h UTC. The 368 nm UVMFRSR channel is not compared to the spectral instruments because the Intercomparison format did not extend out to this wavelength in order to obtain synchronous scans that start on the half hour. The agreement for all three UVMFRSR radiometers with the spectral instruments convolved with the UVMFRSR filter functions is within 7 % except for channel 1 (300 nm nominal wavelength) for USDA_386 and USDA_387. Channel 1 for the USDA_270 instrument agrees with the average within 1 %, but the USDA_386 and USDA_387 instruments are approximately 20 % lower than the average. The filter functions were not determined at the Intercomparison or at the CUCF and possibly the filter transmittances for these channels have changed since their measurement. Ideally, for future Intercomparisons, the filter functions should be determined just prior to the Intercomparison and compared with the previous measurements.

**Fig. 6.7:**
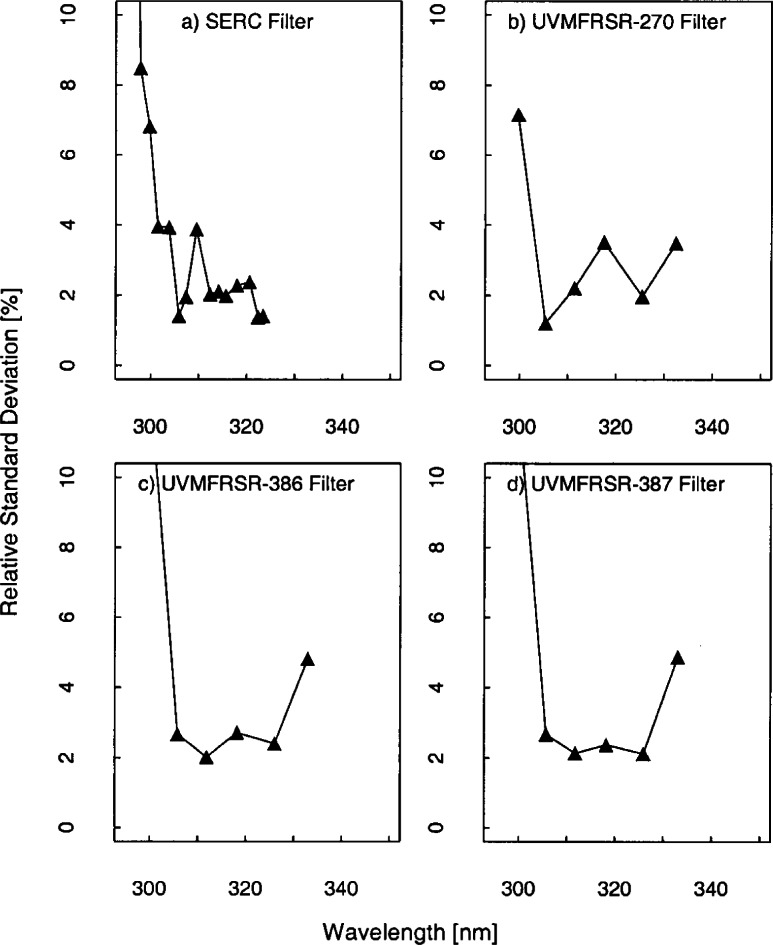
(a)–(d) The relative standard deviation of the solar irradiance at 16.5 h UTC of the spectral instruments and the filter instrument indicated in the panel. The solar irradiance of the spectral instruments is convolved with the filter functions of the instruments indicated in the panel.

Finally, the relative standard deviation for the ASRC_RSS, EPA_101, EPA_114, NSF_SUV, USDA_U1K and SERC instruments convolved with a SERC filter function is less than 4 % for filters greater than 300 nm (Fig. 6.7 a). The relative standard deviations for the ASRC_RSS, EPA_101, EPA_114, NSF_SUV, USDA_U1K and UVMFRSR instruments convolved with the filter functions of the three UVMFRSR instruments, and then compared with these three instruments, is less than 4 % for wavelengths greater than 300 nm (Fig. 6.7 b–d).

## 7. Conclusions

Several prototype and relatively new instruments participated in the 1997 Intercomparison and showed very promising results. The double monochromators of the USDA_U1K and the NSF_SUV instruments and the dual prism of the ASRC_RSS instrument are used to improve stray-light rejection, which can be a significant problem at the shorter UV wavelengths where ozone absorption is the strongest. Accurately measuring solar irradiance at the shorter wavelengths is very desirable because changes in atmospheric ozone concentrations will have a larger impact on the solar irradiance, possibly decreasing the time frame needed for detection of trends. It should be mentioned that the Intercomparison format did not show all the benefits of each instrument. For instance, the ASRC_RSS and the YES_RSS spectrograph instruments, and the UVMFRSRs are capable of measuring all wavelengths simultaneously, and the SERC instrument performs a scan every 4.3 s, which is desirable when investigating a rapidly changing atmosphere such as cloud effects on solar irradiance. These same instruments except the SERC are also rotating shadowband radiometers that are capable of measuring the total irradiance and the diffuse beam, and consequently the direct beam from subtraction. The NSF_SUV instrument is capable of measuring irradiance out to 600 nm, which is useful for many applications in atmospheric research.

As in previous years, the 1997 Intercomparison characterized instruments for stray-light, bandwidth, and wavelength accuracy using spectral lines from a HeCd laser, and Hg, Cd, and Zn lamps. These results are summarized in Table 5.1. The stray-light rejections of the instruments were consistent with those expected for single- and double-grating monochromators and for interference filters. The bandwidths of the EPA_101, EPA_114, and the NIST scanning instruments decreased with increasing wavelength. The bandwidth of the spectrograph instruments, ASRC_RSS and YES_RSS, increased with increasing wavelength. The bandwidth of the NSF_SUV and the USDA_U1K remained relatively constant with wavelength. Many of the newer instruments, ASRC_RSS, NIST, USDA_U1K, and YES_RSS, showed very good wavelength accuracy of better than 0.02 nm. For the EPA instruments, the wavelength uncertainties showed some dependence on wavelength. The wavelength accuracy of the NSF_SUV instrument was less than expected but this is attributable to an error in a wavelength calibration routine that has subsequently been remedied.

The spectral irradiance responsivity of the instruments was determined several times outdoors with the NOAA horizontally calibrated 1000 W lamps using the NOAA field calibrator. The temporal stability of the responsivities is very important for reliability of spectral solar irradiance measurements and should change as little as possible while the instruments are monitoring solar ultraviolet irradiance. There was not sufficient data to completely assess the temporal stability of the instruments under a variety of conditions but the responsivity was determined several times to get an indication of the stability. In this current set of spectroradiometers, the responsivities do not change more than 5 % over 5 days except for the NIST instrument, which had electronic problems related to the weather because the instrument was not weatherproof, and the UVMFRSR_270 which may have had an insufficient warm-up time prior to the measurement. The responsivity of the ASRC_RSS instrument had a negligible offset from one scan to the next but scatter of approximately 10 % at the short and long wavelengths, which is attributed to the low sensitivity of the MOS detector.

Synchronized solar irradiance scans from 290 nm to 360 nm were performed every half-hour during the Intercomparison. Because the instruments had different bandwidths, the measured irradiances were convolved to common bandwidths by using both triangular and Gaussian slit scattering functions and the filter transmittances of the filter-based instruments. The agreement among the convolved irradiances was described by their relative difference from the average. The relative standard deviation in the solar irradiance convolved with a triangular slit-scattering function between the spectral instruments was within 5 % using the corrected NSF_SUV data for wavelengths greater than 300 nm on day 267 at 16.5 h UTC. With the Gaussian slit-scattering function the relative standard deviation was within 4 %. The ASRC_RSS instrument deviates from the average more than expected for 300 nm to 305 nm and the problem becomes more severe as the day progresses. The changes in the relative differences of the spectral instruments with increasing solar zenith angle indicates a possible difference in the angular responses of the instruments, suggesting the need for corrections or improvements in the Lambertian quality of the diffusers and linearity of the detectors. The relative standard deviation of the solar irradiance at 16.5 h UTC including the filter instruments was 4 % for wavelengths above 300 nm. The solar irradiances for filter wavelengths down to 296 nm agree within 10 % to those of the USDA_U1K instrument on day 267 at 16.5 h UTC.

In conclusion, this Intercomparison was successful at characterizing the instruments and comparing the newer instruments with existing UV spectroradiometers. Even with difficult weather, all the instrument characterizations could be performed outdoors. The results from the data yielded valuable information about the performance of the instruments, especially the prototype instruments, and suggested possible improvements in techniques and design.

## Figures and Tables

**Table 1.1:** Instruments present during the 1997 North American Interagency Intercomparison of Ultraviolet Monitoring Spectroradiometers

Agency	Label	Participating spectroradiometers	Serial no.	Purpose
EPA	EPA_101	Sci-Tec Brewer MKIV	101	Monitoring
EPA	EPA_114	Sci-Tec Brewer MKIV	114	Monitoring
NIST	NIST	RSI		Calibration
NSF	NSF_SUV	BSI SUV-150	11-002	Monitoring
SERC	SERC	SERC SR-18	UI	Monitoring
USDA	USDA_U1K	U1000 Spectroradiometer		Monitoring/Calibration
ASRC	ASRC_RSS	UV-RSS Spectrograph	104	Research/Monitoring
USDA	USDA_270	UV-MFRSR	270	Monitoring
USDA	USDA_386	UV-MFRSR	386	Monitoring
USDA	USDA_387	UV-MFRSR	387	Monitoring
YES	YES_RSS	UV-RSS Spectrograph		Research/Monitoring

Ancillary Measurements	

Instrument	Serial no.
Yankee UVB-1 Radiometer	940401
Yankee UVB-1 Radiometer	940402
Yankee UVB-1 Radiometer	940404
Solar Light UV-Biometer	1916
Solar Light UV-Biometer	2004
Solar Light UV-Biometer	2005
Li-Cor Photosynthetically Active Radiation Sensor (PAR)	Q22666
Normal Incidence Pryheliometer (NIP)	30399
Spectrosun Precision Solar Pyranometer (Downwelling)	73–88
Spectrosun Precison Solar Pyranometer (Upwelling)	73–82
Eppley Precison Infrared Pyrgeometer (Downwelling)	31609F3
Eppley Precison Infrared Pyrgeometer (Upwelling)	31608F3
Total Sky Imager (TSI)	

Meteorological Instruments	

Measurement	Instrument
Temperature and Relative Humidty	Vaisala R2410094
Barometric pressure	Vaisala P173002

**Table 3.1:** Spectroradiometer specifications

Participant/Network	EPA_101 EPA_114	NIST	NSF_SUV	SERC	USDA_270 USDA_386 USDA_387	ASRC_RSS	USDA_U1K	YES_RSS
Spectroradiometer Model	Brewer MKIV	RSI	SUV_150	SERC_SR18	UV-MFRSR	UV-RSS^a^	U1000	UV-RSS^a^
Manufacturer	Sci-Tec	NIST	Biospherical Instruments	Smithsonian SERC	Yankee Environmental Systems	SUNY-ASRC	ASRC + Instruments SA	Yankee Environmental Systems
Serial Number	101, 114	Prototype	11-002	UI	280, 386, 387	Prototype, 104	Prototype	Prototype
Scanning/Spectrograph/Filter	Scanning	Scanning	Scanning	Filter	Filter	Spectrograph	Scanning	Spectrograph
Diffraction Grating/Prism	Grating: Modified Ebert Single Monochromator	Grating: Fastie-Ebert Double Monochromator	Grating: Czerny-Turner Double Monochromator	N/A	N/A	Prism: UV-Fused Silica AR coated 2 Prism Spectrograph	Grating: 1-meter Czerny Turner Double Monochromator	Prism: UV-Fused Silica uncoated Double Monochromator
Detector	PMT 9789QA	PMT Hamamatsu R1414	PMT	PMT Hamamatsu R-1657	Silicon Carbide + GaP	CCD 1 × 512	PMT Hamamatsu R2371-02	CCD 256×1024
Nominal Bandwidth [nm]	0.6	0.8	0.7	2	2	0.5	0.1	0.6
Range (nm)	286.5 nm to 363.0 nm	290 nm to 325 nm	280 nm to 600 nm	290 nm to 324 nm in 2 nm steps	300 nm to 368 nm	290 nm to 350 nm	290 nm to 410 nm	295 nm to 345 nm
Diffuser Material	Teflon (4 cm)		Teflon	Teflon (0.75 in)	386-Teflon (7 mm) 387-Teflon 270-Spectralon	Teflon/Spectralon (0.25 in)	PTFE (1 in)	Teflon/Spectralon (0.25 in)
Weatherproof	Yes	No	Yes	Yes	Yes	Yes	Yes	Yes
Temperature stabilized								
Optics	No	No	Yes	No	Yes	Yes	Yes	Yes
Detector	No	No	Yes	Yes	Yes	Yes	Yes	Yes
Filter	<325 nm 0.5 ndf +NiSO4+UG11 >324 1.5 ndf +UG11	None	None	18 Filters, Barr Interference	7 Filters, Barr Interference	NiSO_4_ × 5H_2_0 + VG5		Solar Blind Filter
Dark Current removed	Yes	Yes	Yes	Yes	Yes	Yes	Yes	Yes
Stray Light Removed	Yes	No	Yes	No	No	No	No	No
Wavelength Registration (nm)	302.3		296.7	N/A	N/A			
Primary Lamp (W)	1000W	1000W	200W	1000W	1000W	1000W	1000W	
Secondary Lamp (W)	50W		45W	None	None		20W	

a Rotating Shadowband Radiometer.

**Table 3.2:** Channel indicator, nominal and actual center wavelength, bandwidth, and maximum transmittance for each filter of the SERC SR-18 instrument, serial number UI

Channel	Nominal center wavelength (nm)	Actual center wavelength (nm)	Bandwidth (nm)	Maximum transmittance
I	290	290.00	2.18	0.1124
H	292	291.96	2.08	0.1384
G	294	293.83	2.32	0.1602
F	296	295.87	2.24	0.1076
E	298	297.95	2.22	0.1219
D	300	299.74	2.08	0.1235
C	302	301.53	2.13	0.1313
B	304	303.81	2.63	0.1675
A	306	305.98	2.23	0.1452
J	Dark			
K	308	307.40	2.33	0.1580
L	310	309.61	2.08	0.1985
M	312	312.36	2.09	0.1543
N	314	314.19	2.29	0.1331
O	316	315.69	2.34	0.1447
P	318	317.99	2.47	0.1546
Q	320	320.65	2.61	0.1618
R	322	322.44	2.28	0.1975
S	324	323.42	2.27	0.1997
T	Dark			

**Table 3.3:** Channel indicator, nominal and actual center wavelength, and bandwidth for each filter of USDA instruments 270, 386 AND 387

Channel	Nominal center wavelength (nm)	Actual center wavelength (nm)	Bandwidth (nm)
Unit 270			

0	300	299.73	2.31
1	305	305.42	2.15
2	311	311.47	2.28
3	317	317.65	2.18
4	325	325.48	1.89
5	332	332.46	2.03
6	368	367.78	1.71

Unit 386			

0	300	300.36	2.13
1	305	305.84	2.23
2	311	311.90	2.43
3	317	318.25	2.24
4	325	326.01	1.88
5	332	332.93	2.14
6	368	368.44	1.76

Unit 387			

0	300	300.37	2.17
1	305	305.69	2.23
2	311	311.81	2.39
3	317	318.20	2.20
4	325	325.94	1.85
5	332	333.04	2.13
6	368	368.41	1.81

**Table 5.1:** Summary of stray-light, wavelength accuracy and bandwidth

Participant/Network	EPA_101	EPA_114	NIST	NSF_SUV	SERC	USDA_270 USDA-387 USDA_386	ASRC_RSS	USDA_U1K	YES_RSS
Stray-Light (HeCd) at 300 nm	3 × 10^−5^	6 × 10^−5^	<10^−5^^a^	<10^−6^	4 × 10^−5^		2 × 10^−10^	2 × 10^−10^	
Wavelength Accuracy									
Mean (Offset)	−0.006	−0.025	+0.000	+0.3 (day 260) −0.06 (day 259)			+0.013	+0.016	+0.006
RMS	±0.029	±0.027	±0.012	±0.012 (day 259)			±0.010	±0.007	±0.011
Bandwidth (nm) at 325 nm	0.58	0.58	0.85	0.70	2 nominal	2 nominal	0.53	0.11	0.61

a At 320 nm.

**Table 5.2:** Dates, lamps, times, and instrument temperatures of spectral scans determining responsivity

Instrument	Day	Lamp	Time (UTC)	Instrument temperature (°C)
	266	96599	21.79	19.67
EPA_101	261	96598	2.96	24.51
	263	96599	21.64	15.94
EPA_114	266	96599	21.29	17.8
	260	E002	22.41	15.08
NIST	260	96599	23.29	11.78
	261	96599	0.75	NA
	261	96598	1.60	NA
NSF_SUV	263	96598	18.99	NA
	268	96599	20.90	NA
	268	96598	22.78	NA
	262	96599	0.15	31.82
SERC	263	96599	20.22	19.23
	267	96599	1.38	20.14
	261	96599	1.17	NA
USDA_270	263	96598	13.52	NA
	268	96599	16.4	NA
	261	96598	2.38	NA
USDA_386	263	96598	13.03	NA
	268	96599	17.62	NA
	261	96598	1.82	NA
USDA_387	263	96598	12.85	NA
	268	96599	17.11	NA
	261	96598	NA	NA
ASRC_RSS	264	96598	NA	NA
	268	96599	NA	NA
	261	96598	23.61	NA
USDA_U1K	264	96598	1.02	NA
	268	96599	1.02	NA

**Table 5.3:** Relative standard uncertainties from all components during responsivity measurements at selected wavelengths.

Relative standard uncertainty (%)									
Component	Wavelength (nm)	EPA_101	EPA_114	NIST	NSF_SUV	SERC	USDA_MFRSR (270,386,387)	ASRC_RSS	USDA_U1K
Lamp									
Irradiance									
	290	1.22	1.22	1.22	1.22	1.22	1.02	1.10	1.22
	320	0.91	0.91	0.91	0.91	0.91	0.91	0.91	0.91
Size	350	0.68	0.68	0.68	0.68		0.78	0.83	0.68
Goniometry		0.09	0.09	0.05	0.03	0.02	0.01	0.01	0.05
Current (random)		0.46	0.46	0.36	0.30	0.27	0.11	0.11	0.36
	290	0.06	0.06	0.06	0.06	0.06	0.06	0.06	0.06
	320	0.05	0.05	0.05	0.05	0.05	0.05	0.05	0.05
	350	0.05	0.05	0.05	0.05		0.05	0.05	0.05
Current (systematic)	290	0.11	0.11	0.11	0.11	0.11	0.11	0.11	0.11
	320	0.10	0.10	0.10	0.10	0.10	0.10	0.10	0.10
	350	0.09	0.09	0.09	0.09		0.09	0.09	0.09
Alignment	All	0.39	0.39	0.39	0.39	0.39	0.39	0.86	0.86
Instrument									
Wavelength	290	0.36	0.08	0.04	0.04	0.19	0.18	0.08	0.04
	320	0.11	0.19	0.02	0.15	0.15	0.14	0.00	0.04
	350	0.16	0.04	0.06	0.17		0.11	0.06	0.04
Signal	290	0.82	0.50	0.20	0.56	0.23	(0.13,0.08,0.09)	5.2	0.54
	320	0.49	0.36	1.31	0.28	0.99	(0.28,0.09,0.64)	1.6	0.62
	350	0.47	0.48	1.67	0.19		(0.14,0.07,0.64)	14.8	0.63
Combined									
Random	290	0.82	0.50	0.21	0.56	0.24	(0.14,0.10,0.11)	5.2	0.54
	320	0.49	0.36	1.31	0.28	0.99	(0.28,0.10,0.64)	1.6	0.62
	350	0.47	0.48	1.67	0.20		(0.15,0.09,0.64)	14.8	0.63
Systematic	290	1.41	1.37	1.34	1.32	1.33	1.05	1.40	1.54
	320	1.11	1.12	1.12	0.97	1.04	1.01	1.26	1.31
	350	0.93	0.92	0.87	0.86		0.89	1.20	1.15

**Table 6.1:** Dates and times, indicated by an “X,” at which participating instruments were performing synchronized scans of solar ultraviolet irradiance

Day	Time	EPA_101	EPA_114	NSF_SUV	SERC	ASRC_RSS	USDA_U1K	USDA_270	USDA_386	USDA_387
260	00:00				X			X		
	00:30				X			X		
	01:00				X			X	X	X
	12:00				X			X	X	X
	12:30				X			X	X	X
	13:00			X	X			X	X	X
	13:30				X			X	X	X
	14:00				X		X	X	X	X
	14:30				X		X	X	X	X
	15:00			X	X		X	X		X
	15:30			X	X		X	X	X	X
	16:00				X		X	X	X	X
	16:30			X	X		X	X	X	X
	17:00				X		X	X	X	X
	17:30				X		X	X	X	X
	18:00			X	X		X	X	X	X
	18:30			X	X		X	X	X	X
	19:00			X	X		X	X	X	X
	19:30			X	X		X	X	X	X
	20:00			X	X		X	X	X	X
	20:30				X		X	X	X	X
	21:00								X	X
	21:30								X	X
	22:00								X	X
	22:30								X	X
	23:00								X	X
	23:30								X	X
261	00:00				X					
	00:30				X					
	01:00				X					
	12:00			X	X			X		
	12:30			X	X			X		
	13:00			X	X			X		
	13:30			X	X			X		
	14:00			X	X			X		
	14:30			X				X		
	15:00						X	X		
	15:30			X			X	X		
	16:00			X			X	X		
	16:30			X				X		
	17:00			X				X		
	17:30			X	X			X		
	18:00			X	X		X	X		
	18:30			X	X		X	X		
	19:00			X	X		X	X		
	19:30			X	X			X		
	20:00			X	X			X		
	20:30			X	X			X		
	21:00			X				X		
	21:30			X				X		
	22:00			X				X		
	22:30			X				X		
	23:00			X				X		
	23:30							X		
262	00:00								X	X
	00:30							X	X	X
	01:00							X	X	X
	12:00				X			X	X	X
	12:30				X			X	X	X
	13:00				X			X	X	X
	13:30				X			X	X	X
	14:00				X			X	X	X
	14:30				X		X	X	X	X
	15:00				X			X	X	X
	15:30				X		X	X	X	X
	16:00				X		X	X	X	X
	16:30				X			X	X	X
	17:00				X		X	X	X	X
	17:30				X		X	X	X	X
	18:00				X		X	X	X	X
	18:30				X		X	X	X	X
	19:00				X		X	X	X	X
	19:30				X		X	X	X	X
	20:00				X		X	X	X	X
	20:30				X		X	X	X	X
	21:00				X		X	X	X	X
	21:30				X		X	X	X	X
	22:00				X		X	X	X	X
	22:30				X		X	X	X	X
	23:00				X		X	X	X	X
	23:30				X		X	X	X	X
263	00:00			X	X					
	00:30			X	X			X	X	X
	01:00			X	X			X	X	X
	12:00			X	X			X	X	X
	12:30			X	X			X	X	X
	13:00			X	X			X	X	X
	13:30			X	X			X	X	X
	14:00			X	X			X	X	X
	14:30			X	X			X	X	X
	15:00			X	X			X	X	X
	15:30			X	X			X	X	X
	16:00			X	X			X	X	X
	16:30			X	X			X	X	X
	17:00			X	X			X	X	X
	17:30			X	X	X		X	X	X
	18:00				X	X		X	X	X
	18:30				X	X		X	X	X
	19:00				X	X		X	X	X
	19:30				X	X		X		
	20:00				X			X		
	20:30			X	X	X		X		
	21:00			X	X	X		X		
	21:30			X	X	X		X		X
	22:00			X	X	X			X	
	22:30			X	X	X		X		
	23:00			X	X			X	X	X
	23:30			X	X			X	X	X
264	00:00			X	X			X	X	X
	00:30			X	X			X	X	X
	01:00			X	X			X	X	X
	12:00				X			X	X	X
	12:30				X			X	X	X
	13:00				X			X	X	X
	13:30				X			X	X	X
	14:00				X			X	X	X
	14:30				X			X	X	X
	15:00				X			X	X	X
	15:30				X			X	X	X
	16:00				X			X	X	X
	16:30				X			X	X	X
	17:00				X			X	X	X
	17:30				X			X	X	X
	18:00				X			X	X	X
	18:30				X			X	X	X
	19:00				X	X		X	X	X
	19:30				X	X		X	X	X
	20:00				X	X		X	X	X
	20:30				X	X		X	X	X
	21:00				X	X		X	X	X
	21:30				X	X		X	X	X
	22:00				X			X	X	X
	22:30				X	X		X	X	X
	23:00				X			X	X	X
	23:30				X			X	X	X
265	00:00				X	X		X	X	X
	00:30				X	X		X	X	X
	01:00				X			X	X	X
	12:00				X			X	X	X
	12:30		X		X			X	X	X
	13:00		X		X			X	X	X
	13:30		X		X			X	X	X
	14:00		X		X			X	X	X
	14:30		X		X			X	X	X
	15:00		X		X			X	X	X
	15:30		X		X			X	X	X
	16:00				X			X	X	X
	16:30				X			X	X	X
	17:00				X			X	X	X
	17:30				X	X		X	X	X
	18:00		X		X	X		X	X	X
	18:30				X	X		X	X	X
	19:00		X		X	X		X	X	X
	19:30		X		X	X		X	X	X
	20:00		X		X	X		X	X	X
	20:30		X		X	X		X	X	X
	21:00		X		X	X		X	X	X
	21:30		X		X	X		X	X	X
	22:00		X		X	X		X	X	X
	22:30		X		X	X		X	X	X
	23:00		X		X	X		X	X	X
	23:30		X		X	X		X	X	X
266	00:00		X		X			X	X	X
	00:30		X		X			X	X	X
	01:00		X		X			X	X	X
	12:00		X		X			X	X	X
	12:30		X		X			X	X	X
	13:00		X		X			X	X	X
	13:30		X		X			X	X	X
	14:00		X		X			X	X	X
	14:30		X		X			X	X	X
	15:00		X		X	X		X	X	X
	15:30		X		X	X		X	X	X
	16:00		X		X	X		X	X	X
	16:30		X		X	X		X	X	X
	17:00		X		X	X		X	X	X
	17:30		X		X	X		X	X	X
	18:00		X		X	X		X	X	X
	18:30		X		X	X		X	X	X
	19:00				X	X		X	X	X
	19:30				X	X		X	X	X
	20:00				X	X		X	X	X
	20:30				X	X		X	X	X
	21:00				X	X		X	X	X
	21:30				X	X		X	X	X
	22:00				X			X	X	X
	22:30				X			X	X	X
	23:00				X			X	X	X
	23:30				X			X	X	X
267	00:00				X	X		X	X	X
	00:30			X	X			X	X	X
	01:00			X				X	X	X
	12:00			X				X	X	X
	12:30		X	X				X	X	X
	13:00		X	X				X	X	X
	13:30		X	X				X	X	X
	14:00		X	X				X	X	X
	14:30		X			X		X	X	X
	15:00	X	X	X		X	X	X	X	X
	15:30	X		X		X	X	X	X	X
	16:00	X	X	X	X	X	X	X	X	X
	16:30	X	X	X	X	X	X	X	X	X
	17:00	X	X	X	X	X	X	X	X	X
	17:30	X	X	X	X	X	X	X	X	X
	18:00	X	X	X	X	X	X	X	X	X
	18:30	X	X	X	X	X		X	X	X
	19:00	X	X	X	X	X		X	X	X
	19:30	X	X	X	X	X		X	X	X
	20:00	X	X	X	X	X	X	X	X	X
	20:30	X	X	X	X	X	X	X	X	X
	21:00	X	X	X	X	X	X	X	X	X
	21:30	X	X	X	X	X	X	X	X	X
	22:00	X	X	X	X	X	X	X	X	X
	22:30	X	X	X	X	X	X	X	X	X
	23:00	X	X	X	X	X	X	X	X	X
	23:30		X	X	X	X	X	X	X	X
268	00:00	X		X	X		X	X	X	X
	00:30	X		X	X			X	X	X
	01:00	X		X	X			X	X	X
	12:00				X			X	X	X
	12:30		X		X			X	X	X
	13:00		X		X			X	X	X
	13:30		X		X			X	X	X
	14:00		X		X			X	X	X

**Table 6.2:** Days and times of responsivity scans used to calculate solar irradiance on Julian day 267

Instrument	Day/Time (UTC)	Lamp Number
EPA_101	266/21.8	96599
EPA_114	266/21.3	96599
NSF_SUV	268/20.9	96599
	268/6.2	45W Internal
SERC	267/1.4	96599
USDA_270	268/16.4	96599
USDA_386	268/17.6	96599
USDA_387	268/17.1	96599
ASRC_RSS	263/−	96598
USDA_U1K	266/1.0	96599

## Footnotes

1 Certain commercial equipment, instruments, or materials are identified in this paper to foster understanding. Such identification does not imply recommendation or endorsement by the National Institute of Standards and Technology, nor does it imply that the materials or equipment identified are necessarily the best available for the purpose.

## 8. Appendix A. Attendees

The following people attended the 1997 North American Interagency Intercomparison of Ultraviolet Monitoring Spectroradiometers, and are grouped by function and network.

### Coordinators

**Table:**

National Oceanic and Atmospheric Administration (NOAA)		
John DeLuisi	(303) 497–6083	john.j.deluisi@noaa.gov
Kathleen Lantz	(303) 497–7280	kathy.o.lantz@noaa.gov
Patrick Disterhoft	(303) 497–6355	patrick.disterhoft@noaa.gov
Fax	(303) 497–6546	
NOAA R/E/ARx1		
325 Broadway		
Boulder, CO 80303		

**Table:**

National Institute of Standards and Technology (NIST)		
Ted Early	(301) 975–2343	edward.early@nist.gov
Fax	(301) 840–8551	
NIST		
Bldg. 220, Rm. A-320		
Gaithersburg, MD 20899-0001		

### Participants

**Table:**

National Institute of Standards and Technology (NIST)		
Ambler Thompson	(301) 975–2333	ambler.thompson@nist.gov
Fax	(301) 840–8551	
NIST		
Gaithersburg, MD 20899-0001		

**Table:**

Environmental Protection Agency (EPA)		
Wanfeng Mou		
Thomas Taylor	(706) 542–2492	tetaylor@hal.physast.uga.edu
Fax	(706) 542–2492	
NUVMC/University of Georgia		
Dept. of Physics and Astronomy		
University of Georgia		
Athens, GA 30602		

**Table:**

National Science Foundation (NSF)		
James Ehramjian	(619) 686–1888	uvgroup@biospherical.com
Laura Cabasag	(619) 686–1888	uvgroup@biospherical.com
James Robertson	(619) 686–1888	uvgroup@biospherical.com
Germar Bernhard	(619) 686–1888	uvgroup@biospherical.com
Fax	(619) 686–1887	
Biospherical Instruments, Inc.		
5340 Riley Street		
San Diego, CA 92110-2621		

**Table:**

Smithsonian Environmental Research Center (SERC)	
Doug Hayes	
Fax	(301) 261–7954
Smithsonian Environmental Research Center	
P.O. Box 28	
Edgewater, MD 21037	

**Table:**

United States Department of Agriculture (USDA)		
Lee Harrison	(518) 437–8741	lee@asrc.cestm.albany.edu
Jerry Berndt	(518) 437–8738	jerry@asrc.cestm.albany.edu
Peter Kiedron	(518) 437–8737	
	kiedron@asrc.cestm.albany.edu	
Fax	(518) 437–8758	
Atmospheric Sciences Research Center		
State University of New York at Albany		
CESTM Building		
251 Fuller Road		
Albany, NY 12205		

**Table:**

United States Department of Agriculture (USDA)		
James Slusser	(970) 491–3623	sluss@nrel.colstate.edu
David Bigelow	contact James Slusser	
Bill Durham	(970) 491–3604	billd@nrel.colostate.edu
George Janson	(970) 491–3621	georgej@nrel.colstate.edu
Natural Resource Ecology Laboratory		
Colorado State University		
Fort Collins, CO 90523		

**Table:**

Yankee Environmental Systems, Inc. (YES_RSS)		
Arthur Beaubien	(413) 863–0200	afb@yesinc.com
Mark Beaubien	(413) 863–0200	mcb@yesinc.com
Airport Industrial Park		
Turner Falls, MA 01376		

## 9. Appendix B. Responsivity Uncertainty Analysis

A general description of the uncertainty analysis has been given previously [5]. The following is the specific information used to determine the uncertainties in the responsivity measurement for the prototype instruments. The specific information on instruments that had attended previous Intercomparisons is given in Ref. [5]. The sizes of the diffusers are parameters for the uncertainties arising from both the distribution of flux over the diffuser and the goniometric distribution of flux from the lamp (*g*). The radii of the diffusers of the instruments are given in Table B.1. For the goniometric distribution, *g*_avg_ = 0.995 was assumed for both lamps, and the distance *D* = 50 cm.

**Table B.1:** Diffuser Radii

Instrument	Radius (cm)
EPA_101	1.6
EPA_114	1.6
NIST	1.27
NSF_SUV	1.05
SERC	0.95
USDA_270,386,387	0.4
ASRC_RSS	0.4
USDA_U1K	1.27
YES_RSS	0.4

The quadrature sum of the standard uncertainty in lamp current arising from random effects is 0.42 mA. The quadrature sum of the standard uncertainty in current arising from systematic effects is 0.81 mA. For the lamp goniometric distribution, *g*_max_ = 0.01 was assumed for both lamps. The uncertainty in the retro-reflected position of the laser beam from the lamp jig was 0.2 cm, the positioning uncertainty in centering the beam on the diffuser was 0.1 cm for the EPA instruments, the SERC, UV-MFRSRs and NIST instruments and 0.2 cm for the others. The uncertainty in centering the beam on the lamp jig was 0.16 cm for the EPA instruments, NIST, NSF_SUV, the UV-MFRSRs, and SERC and 1.0 for the others. The uncertainty in the distance between the diffuser and the lamp jig was 0.2 cm for the ASRC_RSS, USDA_U1K, and YES_RSS and 0.1 cm for the others. When the field calibration unit was used to mount the lamp, the uncertainty in aligning the lamp jig perpendicular to the optic axis was 0.5°, the uncertainty in centering the lamp jig was 0.1 cm, and the uncertainty in the distance between the lamp jig and the diffuser was 0.1 cm.

The uncertainty in wavelength was determined by fitting the centroid differences shown in Fig. 5.4 with a second order polynomial. The fit is appropriate for the given wavelength range. The wavelength uncertainty for the SERC instrument was determined during the 1995 Intercomparison [5]. The coefficients for the second order polynomial fits are given in Table B.2.

**Table B.2:** Wavelength uncertainties

Instrument	Wavelength range (nm)	Wavelength uncertainties		Coefficient, *c*_2_
		Coefficient, *c*_0_	Coefficient, *c*_1_	
EPA_101	290 to 350	8.99E+00	−5.61E–02	8.71E–05
EPA_114	290 to 350	7.78E+00	−4.78E–02	7.32E–05
NIST	230 to 325	−7.12E–01	5.06E–03	−8.85E–06
NSF_SUV	290 to 450	9E–02	0	0
SERC	290 to 324	5E–02	0	0
USDA_270,386,387	300 to 368	5E–02	0	0
ASRC_RSS	290 to 350	3.66E+00	−2.28E–02	3.55E–05
USDA_U1K	290 to 400	−1.14E+00	2.91E–03	−4.13E–06
YES_RSS	290 to 350	−1.14E+00	7.62E–03	−1.26E–05
